# TUT7 controls the fate of precursor microRNAs by using three different uridylation mechanisms

**DOI:** 10.15252/embj.201590931

**Published:** 2015-05-15

**Authors:** Boseon Kim, Minju Ha, Luuk Loeff, Hyeshik Chang, Dhirendra K Simanshu, Sisi Li, Mohamed Fareh, Dinshaw J Patel, Chirlmin Joo, V Narry Kim

**Affiliations:** 1 Center for RNA Research, Institute for Basic ScienceSeoul, Korea; 2 School of Biological Sciences, Seoul National UniversitySeoul, Korea; 3 Kavli Institute of NanoScience, Department of BioNanoScience, Delft University of TechnologyDelft, The Netherlands; 4 Structural Biology Program, Memorial Sloan-Kettering Cancer CenterNew York, NY, USA

**Keywords:** precursor microRNA, single-molecule fluorescence, TUT4 (ZCCHC11), TUT7 (ZCCHC6), uridylation

## Abstract

Terminal uridylyl transferases (TUTs) function as integral regulators of microRNA (miRNA) biogenesis. Using biochemistry, single-molecule, and deep sequencing techniques, we here investigate the mechanism by which human TUT7 (also known as ZCCHC6) recognizes and uridylates precursor miRNAs (pre-miRNAs) in the absence of Lin28. We find that the overhang of a pre-miRNA is the key structural element that is recognized by TUT7 and its paralogues, TUT4 (ZCCHC11) and TUT2 (GLD2/PAPD4). For group II pre-miRNAs, which have a 1-nt 3′ overhang, TUT7 restores the canonical end structure (2-nt 3′ overhang) through mono-uridylation, thereby promoting miRNA biogenesis. For pre-miRNAs where the 3′ end is further recessed into the stem (as in 3′ trimmed pre-miRNAs), TUT7 generates an oligo-U tail that leads to degradation. In contrast to Lin28-stimulated oligo-uridylation, which is processive, a distributive mode is employed by TUT7 for both mono- and oligo-uridylation in the absence of Lin28. The overhang length dictates the frequency (but not duration) of the TUT7-RNA interaction, thus explaining how TUT7 differentiates pre-miRNA species with different overhangs. Our study reveals dual roles and mechanisms of uridylation in repair and removal of defective pre-miRNAs.

## Introduction

MicroRNAs (miRNAs) are generated by multiple maturation steps that consist of two endonucleolytic reactions (Ha & Kim, 2014). First, the nuclear RNase III Drosha cleaves a primary miRNA transcript (pri-miRNA) and releases a ∼70-nt hairpin-shaped RNA (pre-miRNA) with a 2-nt 3′ overhang (Lee *et al*, 2003). The pre-miRNA is exported to the cytoplasm by exportin 5 (Yi *et al*, 2003; Bohnsack *et al*, 2004; Lund *et al*, 2004) and is processed by another RNase III Dicer into a mature miRNA duplex (Bernstein *et al*, 2001; Grishok *et al*, 2001; Hutvagner *et al*, 2001; Ketting *et al*, 2001; Knight & Bass, 2001). The mature miRNA duplex is loaded onto an Argonaute (Ago) protein to form an effector complex called RNA-induced silencing complex (RISC) (Mourelatos *et al*, 2002; Kawamata & Tomari, 2010).

In addition to the canonical miRNA biogenesis pathway, non-canonical cleavage of pre-miRNA has been reported (Yang & Lai, 2011; Ha & Kim, 2014). Pre-miRNAs are often heterogeneous at their 3′ ends, indicating that they are cleaved or trimmed after Drosha processing (Newman *et al*, 2011; Burroughs *et al*, 2012; Heo *et al*, 2012; Li *et al*, 2013). Ago2 contributes to the production of truncated species by cleaving pre-miRNAs in the middle of the 3′ strand. This results in truncated hairpins called Ago-cleaved pre-miRNAs (ac-pre-miRNAs) (Diederichs & Haber, 2007). There is little evidence that ac-pre-miRNAs generate mature miRNAs with an exception of ac-pre-miR-451 that is shorter than others and trimmed further into mature miRNA (Cheloufi *et al*, 2010; Cifuentes *et al*, 2010; Yang *et al*, 2010; Yoda *et al*, 2013). Thus, it remains unclear whether ac-pre-miRNAs have a certain biological role in general or whether they are mostly degradation intermediates. Additional nucleases have been reported to cleave pre-miRNAs (Suzuki *et al*, 2011; Upton *et al*, 2012; Asada *et al*, 2014). However, the molecular mechanism of how the truncated pre-miRNAs are removed is largely unknown.

Accumulating evidence indicates the importance of RNA tailing in the control of RNA stability and function (Ji & Chen, 2012; Ameres & Zamore, 2013; Norbury, 2013; Scott & Norbury, 2013). Uridylation is one of the most frequent types of RNA tailing that occurs on diverse RNA species including miRNAs and mRNAs (Li *et al*, 2005; Scott & Norbury, 2013; Chang *et al*, 2014; Lee *et al*, 2014; Lim *et al*, 2014). Uridylation is carried out by a group of non-canonical poly(A) polymerases (PAPs), also called terminal uridylyl transferases (TUTases or TUTs), which belong to DNA polymerase β superfamily (Aravind & Koonin, 1999). TUTs are conserved throughout most eukaryotes (Stevenson & Norbury, 2006; Martin & Keller, 2007; Wilusz & Wilusz, 2008; Norbury, 2013; Scott & Norbury, 2013). Seven TUTs with distinct substrate specificity, localization, and functions have been described in humans.

Recent studies have revealed that TUT4 (also known as ZCCHC11), TUT7 (ZCCHC6), and TUT2 (GLD2/PAPD4) play crucial roles in let-7 miRNA biogenesis in mammals (Heo *et al*, 2012). In embryonic stem cells and cancer cells, TUT4 and TUT7 (TUT4/7) have been shown to oligo-uridylate precursors of let-7 family miRNAs in concert with the processivity factor Lin28 (Heo *et al*, 2008, 2009; Hagan *et al*, 2009; Yeom *et al*, 2011; Thornton *et al*, 2012). The oligo-U tail inhibits pre-miRNA processing by Dicer and promotes degradation by 3′ to 5′ exonuclease DIS3L2 (Heo *et al*, 2008; Chang *et al*, 2013; Ustianenko *et al*, 2013). In contrast, in somatic cells where Lin28 is not expressed, TUT7, TUT4, and TUT2 (TUT7/4/2) mono-uridylate group II pre-miRNAs redundantly to enhance Dicer processing (Heo *et al*, 2012). Unlike prototypical group I pre-miRNAs which have an optimal 2-nt 3′ overhang for Dicer processing, group II pre-miRNAs have a shorter and defective overhang (1-nt 3′) due to a conserved bulge at the Drosha cleavage site. Mono-uridylation by TUT7/4/2 restores the optimal 2-nt 3′ overhang of group II pre-miRNAs resulting in efficient Dicer processing (Heo *et al*, 2012). Between the two contrasting roles that TUTs play in miRNA biogenesis, Lin28-dependent oligo-uridylation by TUT4/7 has been intensively characterized via biochemical and structural studies (Heo *et al*, 2008, 2009; Hagan *et al*, 2009; Nam *et al*, 2011; Yeom *et al*, 2011; Loughlin *et al*, 2012; Mayr *et al*, 2012; Thornton *et al*, 2012), whereas mechanism of mono-uridylation has been largely unknown.

In this study, we delineate the molecular mechanism of uridylation of pre-miRNAs with various structures. By mapping out the interactions between TUT7 and pre-miRNA, we show that the overhang of a pre-miRNA is the key structural element that TUT7 recognizes. Sensing the overhang structure, TUT7 preferentially uridylates 3′ truncated pre-miRNAs as well as group II pre-miRNA. Uridylation leads to two opposing consequences. Mono-uridylation of intact group II pre-let-7s (with a 1-nt 3′ overhang) restores functional pre-miRNAs (with a 2-nt 3′ overhang). On the contrary, recognition of pre-miRNAs with 5′ overhang (ac-pre-miRNA or trimmed decay intermediates) leads to oligo-uridylation and RNA degradation. Our single-molecule study further reveals that TUT7 employs a distributive mode for both uridylation pathways and that TUT7 discriminates its substrates by interacting with them at different frequencies.

## Results

### TUT7 domains required for mono-uridylation

To map the interactions between TUTs and pre-miRNA, we first sought to identify the minimal domains used for pre-miRNA recognition and mono-uridylation. TUTs share a common catalytic motif consisting of a nucleotidyl transferase (Ntr) and the poly(A) polymerase-associated (PAP) domain (Kwak & Wickens, 2007; Martin & Keller, 2007). The Ntr domain contains three catalytic aspartates, whereas the PAP domain provides nucleotide specificity through its contact with the base in the active site (Martin & Keller, 2007; Lunde *et al*, 2012; Munoz-Tello *et al*, 2012; Yates *et al*, 2012). While TUT2 has only one catalytic motif, TUT7 and TUT4 (TUT7/4) have a duplication of the catalytic motif at their N-terminus although it is inactive due to the lack of one of the catalytic aspartates (Supplementary Fig S1A). Additionally, TUT7/4 possesses a CCHH zinc finger motif at their N-terminus and three CCHC zinc finger motifs around the catalytic motif.

**Figure 1:**
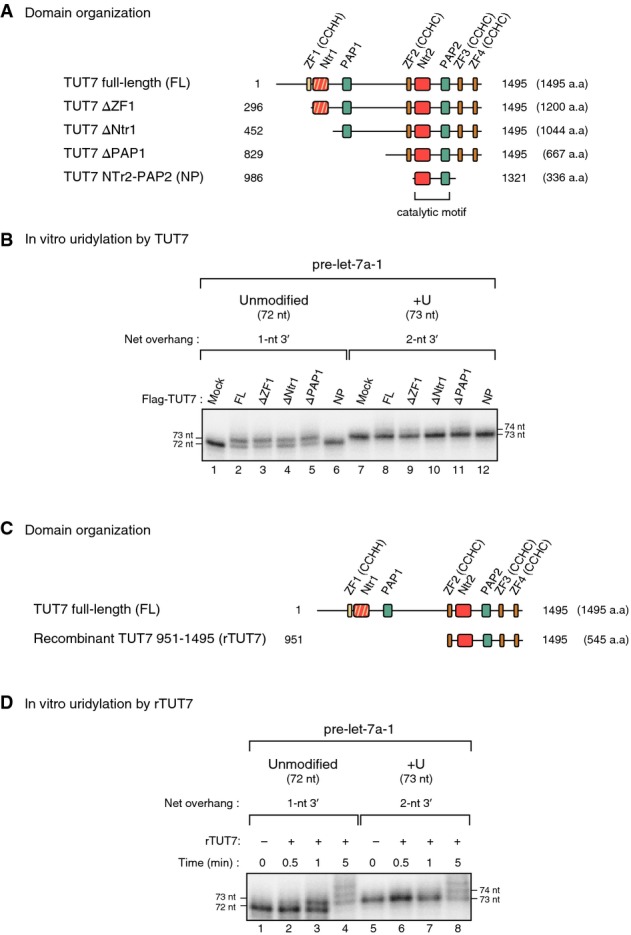
C-terminal half of TUT7 is sufficient to mono-uridylate pre-let-7a-1 specifically

We focused on TUT7 as it is the major enzyme for pre-let-7 mono-uridylation (Heo *et al*, 2012). We generated three deletion mutants of TUT7 by deleting domains from the N-terminus of TUT7 (ΔZF1, ΔNtr1, and ΔPAP1) (Fig1A). In addition, we produced Ntr2-PAP2 (NP) mutant that consists of only the active catalytic motif. The truncated proteins were immunopurified and incubated with unmodified pre-let-7a-1 (with a 1-nt 3′ overhang) or its mono-uridylated counterpart (+U, with a 2-nt 3′ overhang) (Fig1B). The ΔZF1, ΔNtr1, and ΔPAP1 mutants mono-uridylated unmodified pre-let-7a-1 selectively and as efficiently as the full-length (FL) TUT7. However, NP mutant did not show any detectable activity in spite of its higher expression level than that of full-length TUT7 (Fig1B and Supplementary Fig S1B). It seems that NP mutant cannot uridylate RNA substrates, possibly because surrounding regions of catalytic motif are required to bind RNA and/or to maintain proper protein structure. These results indicate that the N-terminal half of TUT7 is dispensable while the C-terminal domains including the catalytic motif are required for pre-miRNA mono-uridylation. As the C-terminal half of TUT7 (ΔPAP1) is fully active, we generated recombinant TUT7 (rTUT7) encompassing 951–1,495 a.a (Fig1C) (Lim *et al*, 2014). *In vitro* uridylation assays demonstrated that rTUT7 can mono-uridylate pre-let-7a-1 and that it has the same substrate preference as the immunopurified full-length TUT7 does (Fig1D). This suggests that the residues 951–1,495 are sufficient for mono-uridylation activity by TUT7. Note that prolonged incubation leads to ‘oligo’-uridylation (Fig1D, lanes 4 and 8) due to multiple rounds of distributive ‘mono’-uridylation (see Discussion for further explanation). Given that the recombinant protein was produced in *E. coli* and purified to homogeneity, our result indicates that TUT7 does not require any additional cofactors for pre-miRNA mono-uridylation (Fig1B and D).

Domain organization of full-length (FL) and deletion mutants (ΔZF1, ΔNtr1, ΔPAP1, and NP) of human TUT7. Yellow, CCHH-type zinc finger; hatched red, inactive nucleotidyl transferase domain due to a sequence variation; green, PAP-associated domain; orange, CCHC-type zinc finger; red, nucleotidyl transferase domain.

*In vitro* uridylation of unmodified pre-let-7a-1 and mono-uridylated pre-let-7a-1 (+U) by immunopurified full-length TUT7 and deletion mutants (15 min reaction). Deletion mutants except for NP showed mono-uridylation activity and the same substrate preference as that of full-length TUT7; they mono-uridylate unmodified pre-let-7a-1 with a 1-nt 3′ overhang more efficiently than pre-let-7a-1 +U with a 2-nt 3′ overhang. NP mutant lost its uridylation activity.

Domain organization of recombinant TUT7 951–1,495 (rTUT7).

*In vitro* uridylation of unmodified pre-let-7a-1 and +U by rTUT7. rTUT7 exhibited mono-uridylation activity and the same substrate preference as full-length TUT7.

Source data are available online for this figure.

### RNA motifs that are recognized by TUT7

**Figure 2:**
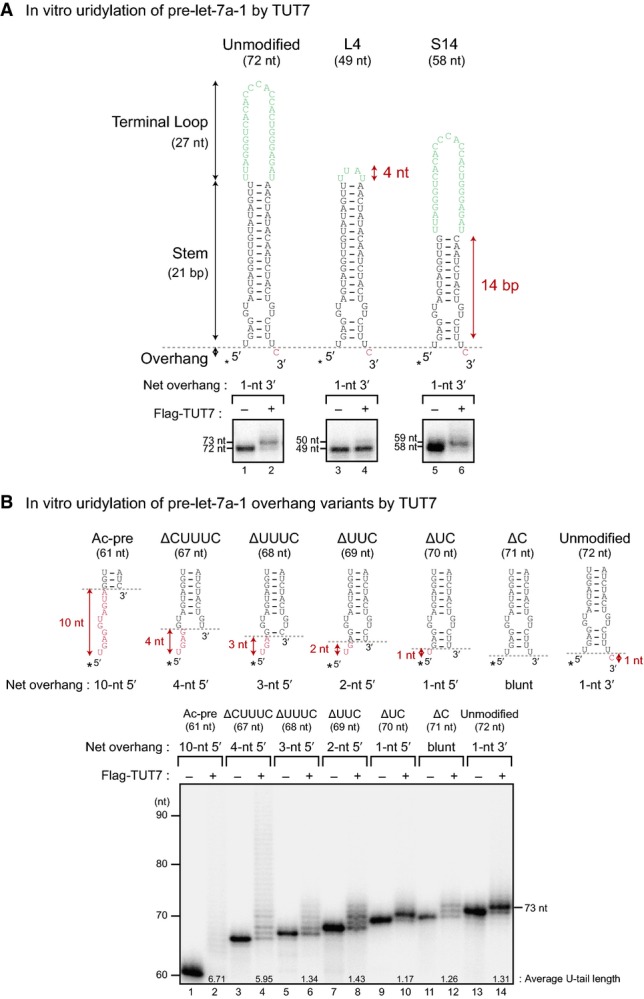
TUT7 recognizes overhang and terminal loop of pre-let-7a-1

To investigate which parts of pre-miRNA are recognized by TUT7, we generated mutants of pre-let-7a-1, a pre-miRNA that belongs to group II. Pre-let-7a-1 is divided up into three parts; a 27-nt terminal loop (green), a 21-bp base-paired stem (black), and a 1-nt 3′ overhang (red) (Fig2A, left). First, we designed a terminal loop mutant (L4) by reducing the loop size from 27 nt to 4 nt (Fig2A, center). Immunopurified full-length TUT7 failed to uridylate the L4 mutant efficiently, which suggested that TUT7 recognizes the terminal loop for mono-uridylation (Fig2A, lanes 1–4). Next, to test whether a stem of a certain length is necessary, we generated a stem mutant (S14) by shortening the stem from 21 bp to 14 bp (Fig2A, right). The S14 mutant was uridylated as efficiently as the unmodified pre-let-7a-1, indicating that the overall length of the stem is not critical for pre-miRNA mono-uridylation by TUT7 (Fig2A, lanes 1–2 and 5–6).

Top: Structure of unmodified pre-let-7a-1, terminal loop mutant (L4), and stem mutant (S14). Green, terminal loop; black, stem; red, overhang. Asterisks mark radio-labeled terminal phosphates. Red arrows indicate the length of the terminal loop or the stem of pre-let-7a-1 L4 or S14, respectively. Bottom: *In vitro* uridylation of unmodified pre-let-7a-1 and mutants by immunopurified full-length TUT7 (13 min reaction). Ablating the terminal loop (L4) dramatically reduced the mono-uridylation efficiency, while shortening the stem (S14) did not affect the mono-uridylation efficiency.

Top: Structure of unmodified pre-let-7a-1 and six overhang variants. Red, overhang. Asterisks mark radio-labeled terminal phosphates, and red arrows indicate the net length of overhang. Bottom: *In vitro* uridylation of unmodified pre-let-7a-1 and overhang variants by immunopurified full-length TUT7 (15 min reaction). TUT7 uridylated blunt end or 5′ overhang variants efficiently and showed enhanced activity to the long 5′ overhang variants (ΔCUUUC and Ac-pre). The average length of U-tail is shown below each band. See Materials and Methods for quantification method.

Source data are available online for this figure.

To find out how the overhang structure influences the mono-uridylation activity of TUT7, we designed six overhang variants of pre-let-7a-1 by shortening nucleotides from the 3′ end (Fig2B, top). We included ac-pre-let-7a-1 (Ac-pre) with a 10-nt 5′ overhang, which is known to be uridylated in humans and mice (Diederichs & Haber, 2007; Newman *et al*, 2011; Burroughs *et al*, 2012; Li *et al*, 2013). The substrates with a blunt end or a 5′ overhang were uridylated with comparable efficiency to (if not more efficiently than) unmodified pre-let-7a-1 by immunopurified full-length TUT7 (Fig2B, bottom). To our surprise, RNAs containing a long 5′ overhang (ΔCUUUC and Ac-pre) were strongly oligo-uridylated. Similar results were obtained with rTUT7 951–1,495 (Supplementary Fig S2A and B). These data indicate that TUT7 acts efficiently on 3′ truncated pre-miRNAs in the absence of any cofactor.

As TUT7/4/2 can act redundantly to mono-uridylate group II pre-let-7s (Heo *et al*, 2012), we also performed *in vitro* uridylation using immunopurified full-length TUT4 and TUT2 (TUT4/2) to compare their substrate preferences. By and large, TUT7/4/2 are highly similar to each other in specificity but they also displayed some distinct characteristics (Fig2 and Supplementary Fig S2C and D). For example, unlike TUT7, TUT4/2 uridylated the terminal loop mutant (L4) as efficiently as unmodified pre-let-7a-1, indicating that TUT4/2 do not interact with the terminal loop for mono-uridylation (Fig2A and Supplementary Fig S2C). Moreover, while TUT7 and TUT4 showed a strong oligo-uridylation activity on pre-miRNAs with a long 5′ overhang (pre-let-7a-1 ΔCUUUC and Ac-pre), TUT2 did not show such activity (Fig2B and Supplementary Fig S2D). Taken together, the primary cis-acting element recognized commonly by TUT7/4/2 is the overhang structure of pre-miRNA.

### Differentiation of pre-miRNAs at the binding step

**Figure 3:**
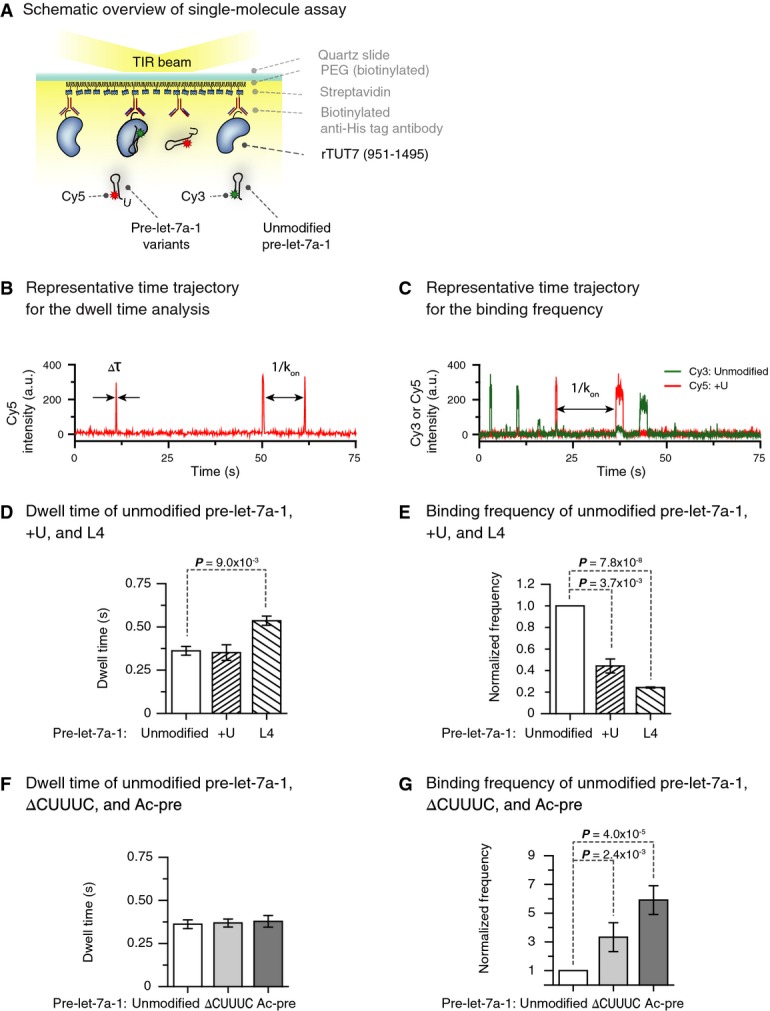
TUT7 distinguishes pre-miRNA substrates at binding step

To further investigate the molecular mechanism by which TUT7 recognizes structural elements of its substrates, we employed single-molecule fluorescence spectroscopy. For long-term single-molecule observations, rTUT7 fused to a 6×-His tag was immobilized on a PEGylated quartz surface using anti-His tag antibodies (Fig3A). Fluorescently labeled pre-let-7a-1 molecules were introduced to the microfluidic chamber, and the interactions between TUT7 and RNAs were monitored in real time. As shown in representative time traces (Fig3B and C), the interaction of rTUT7 with RNA molecules was marked with a sudden increase and subsequent rapid decrease in the fluorescence intensity. This brief interaction suggests that uridylation by TUT7 is distributive. This is similar to Lin28-independent mono-uridylation by TUT4, which we previously reported to be distributive (Yeom *et al*, 2011). Control experiments showed that neither dye-labeling of pre-let-7a-1 nor immobilization of rTUT7 affected its uridylation efficiency (unpublished observations).

Schematic overview of the single-molecule assay. Recombinant TUT7 951–1,495 (rTUT7) fused to a 6×-His was immobilized on a PEGylated surface using anti-His tag antibodies. Afterward, fluorescently labeled RNA substrates were introduced into the chamber.

Representative time trajectory for the dwell time analysis. Δτ, dwell time of interaction; k_on_, binding rate.

Representative time trajectory for binding frequency measurements. k_on_, binding rate.

Average dwell time of pre-let-7a-1 +U and L4 mutants (*n* = 3). Pre-let-7a-1 +U showed similar dwell time to unmodified pre-let-7a-1. Pre-let-7a-1 L4 yielded slightly increased dwell time (*P*-value 9.0 × 10^−3^, two-tailed *t*-test). Error bars represent standard error.

Binding frequency of pre-let-7 +U and L4 mutants relative to unmodified pre-let-7a-1 (*n* = 3). Pre-let-7a-1 +U and L4 mutants showed much lower binding frequency compared to unmodified pre-let-7a-1 (*P*-values 3.7 × 10^−3^ and 7.8 × 10^−8^, respectively, two-tailed *t*-test). Error bars represent standard error.

Average dwell time of pre-let-7a-1 ΔCUUUC and Ac-pre mutants (*n* = 3). Pre-let-7a-1 ΔCUUUC and Ac-pre had similar dwell times compared to unmodified pre-let-7a-1. Error bars represent standard error.

Binding frequency of pre-let-7 ΔCUUUC and Ac-pre mutants relative to unmodified pre-let-7a-1 (*n* = 3). Pre-let-7a-1 ΔCUUUC and Ac-pre mutants displayed much higher binding frequency compared to unmodified pre-let-7a-1 (*P*-values 2.4 × 10^−3^ and 4.0 × 10^−5^, respectively, two-tailed *t*-test). Error bars represent standard error.

Data information: All data sets (D–G) are normally distributed (Shapiro–Wilk test, *P *> 0.1). All the datasets of the binding frequency measurements showed equality in variance (*F*-test). For the dwell time measurements, some data sets did not show equality in variance (+U and -CUUUC) in *F*-test, and we have adjusted our two-tailed *t*-tests accordingly.

Using this experimental system, we questioned at which kinetic step TUT7 discriminates between different RNA substrates. We first determined the dissociation rate (k_off_) by analyzing the dwell time of interaction (Δτ, the inverse of k_off_) between TUT7 and unmodified pre-let-7a-1. The dwell time distribution from a total of 8,943 binding events followed a single-exponential decay with a time scale of 0.36 ± 0.03 s (Supplementary Fig S3A). This indicates that dissociation of an RNA substrate from TUT7 is a single-step process. To gain more insights into the molecular mechanism of TUT7, we repeated this measurement for mono-uridylated pre-let-7a-1 (+U) and the terminal loop mutant (L4). Intriguingly, the dwell time of the +U substrate (0.35 ± 0.05 s, 17,003 events; Fig3D) was similar to that of unmodified substrate within an error, whereas that of the terminal loop mutant showed a slight increase (0.54 ± 0.03 s, 8,012 events; Fig3D). These results suggest that the binding strength between TUT7 and RNA (Supplementary Fig S3B) is not a dominant factor in distinguishing between different RNA substrates, although the terminal loop might play a role in the release of the substrate.

Next, we asked whether the binding rate (k_on_) might govern the substrate preference of TUT7. For this measurement, unmodified pre-let-7a-1 and a variant (e.g. +U) were labeled with spectrally separated fluorescent dyes (Cy3 and Cy5, respectively) and introduced together into a microfluidic chamber. Unmodified pre-let-7a-1 served as a reference. By monitoring the interactions between immobilized rTUT7 and two RNA substrates simultaneously, we were able to compare k_on_ of a variant to that of unmodified pre-let-7a-1 (Fig3A and C). This frequency measurement revealed that the mono-uridylated substrate (+U) binds less frequently than unmodified substrate does (k^+U^_on_/k^Unmodified^_on_ = 0.44 ± 0.07, Fig3E), which indicates that the addition of a single uridine suppresses the TUT7-RNA interaction. Stronger suppression was observed with the terminal loop mutant (L4) (k^L4^_on_/k^Unmodified^_on_ = 0.24 ± 0.01). This is consistent with the decrease in uridylation efficiency observed in Fig2A. Taken together, TUT7 may discriminate between the substrates during the binding step rather than after binding to RNA (Supplementary Fig S3B).

Our biochemical study (Fig2B) indicated that TUT7 oligo-uridylates truncated pre-let-7a-1 effectively under the condition where unmodified pre-let-7a-1 is mono-uridylated. We questioned whether uridylation is changed from a distributive to a processive mode in the presence of the 5′ overhang, or whether the 5′ overhang increases the frequency of the distributive interaction. We repeated our single-molecule kinetic measurements for two pre-let-7a-1 mutants with different 5′ overhang (ΔCUUUC with 4-nt 5′ overhang and Ac-pre with 10-nt 5′ overhang). Intriguingly, the dwell times (Δτ = 1/k_off_) of pre-let-7a-1 ΔCUUUC and Ac-pre were within the error comparable to that of unmodified pre-let-7a-1 (ΔCUUUC, 0.37 ± 0.02 s, 3,495 events; Ac-pre, 0.38 ± 0.03 s, 10,308 events; Fig3F). This suggests that the uridylation mode remains distributive (Fig3F). Thus, in the absence of the processivity factor Lin28, TUT7 exclusively uses a distributive mode for both mono-uridylation and oligo-uridylation.

We next assessed k_on_ and compared the binding frequency of these RNAs with that of unmodified pre-let-7a-1. Indeed, TUT7 interacted with ΔCUUUC and Ac-pre substrates with a higher frequency than with the unmodified substrate (k^ΔCUUUC^_on_/k^Unmodified^_on_ = 3.33 ± 1.01, k^Ac-pre^_on_/k^Unmodified^_on_ = 5.91 ± 1.00; Fig3G). Moreover, binding frequency increased as the length of 5′ overhang got longer (from 4 nt to 10 nt). These results collectively hint that TUT7 distinguishes between its substrates at the binding step.

We further validated the distributive mode of TUT by an *in vitro* uridylation assay with dilution. The reaction mixture with recombinant TUT4 267–1,312 (rTUT4, Supplementary Fig S4A andB) and RNA (unmodified pre-let-7a-1 or ac-pre-let-7a-1) was either not diluted or diluted four times with reaction buffer after 20 s, which should lower the uridylation efficiency in case of distributive uridylation. As a control for processive reaction, uridylation assay with rTUT4 and rLin28b was included. Of note, we used rTUT4 (267–1,312) instead of rTUT7 (951–1,495) because rTUT4 interacts with Lin28, while rTUT7 lacks the first zinc finger motif known to mediate the interaction with Lin28 (Thornton *et al*, 2012). We failed to produce soluble full-length rTUT7 protein. In the presence of Lin28b, rTUT4 oligo-uridylated unmodified pre-let-7a-1, which was not affected by dilution (Supplementary Fig S4C, lanes 7–8). This is consistent with our previous single-molecule data that Lin28-mediated oligo-uridylation is a processive reaction (Yeom *et al*, 2011). In contrast, oligo-uridylation of ac-pre-let-7a-1 (Supplementary Fig S4C, lanes 1–3) and mono-uridylation of unmodified pre-let-7a-1 (Supplementary Fig S4C, lanes 4–6) was strongly reduced after dilution (Supplementary Fig S4C, lanes 1–6). This result suggests that TUTases act distributively in the absence of Lin28, supporting our conclusion from single-molecule measurements (Fig3).

### Uridylation of 3′ trimmed pre-miRNAs in cells

**Figure 4:**
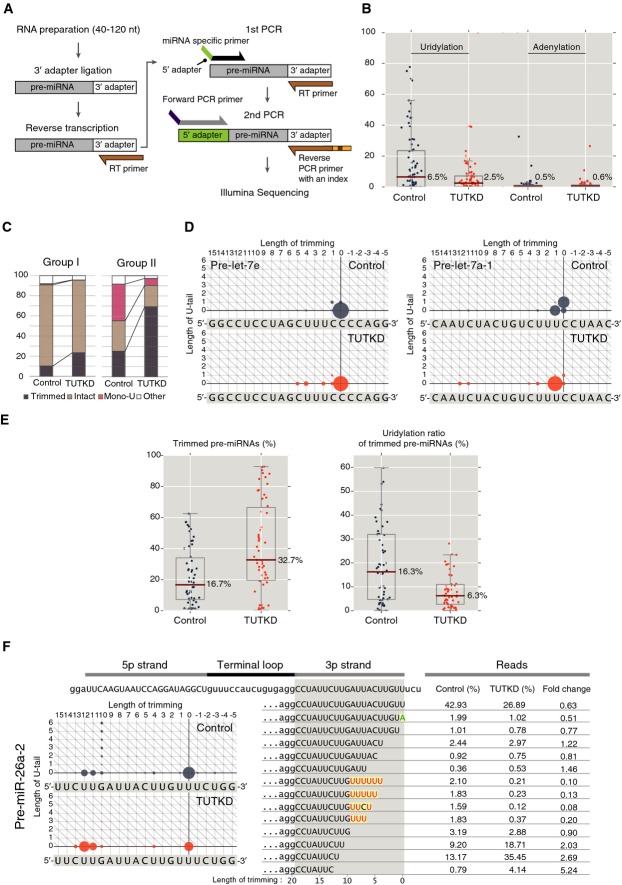
3′ trimmed pre-miRNAs are uridylated by TUT7/4/2 *in vivo*

It is interesting that TUT7 is capable of oligo-uridylating pre-miRNAs with a blunt end or 5′ overhangs *in vitro* (Fig2B). To investigate whether the 3′ truncated pre-miRNAs are indeed uridylated by TUT7 in cells, we carried out pre-miRNA deep sequencing in HeLa cells with or without TUT7/4/2 knockdown (Fig4A and Supplementary Fig S5). We depleted TUT7, TUT4, and TUT2 simultaneously due to their redundant activities (Heo *et al*, 2012). Pre-miRNA library was constructed by size fractionation, 3′ adapter ligation, reverse transcription followed by PCR using primers specific to pre-miRNAs (Fig4A). We selected 55 pre-miRNAs whose miRNAs are abundant in HeLa cells and/or those reported to produce ac-pre-miRNAs (Diederichs & Haber, 2007; Burroughs *et al*, 2012; Li *et al*, 2013) (Supplementary Table S1).

Scheme of pre-miRNA deep sequencing.

Box plot of uridylation ratio and adenylation ratio of 78 pre-miRNAs, which are sequenced in both control sample and TUT7/4/2 knockdown cells. Dark red line indicates the median. TUT7/4/2 knockdown suppressed uridylation specifically.

Mono-uridylation of group I (let-7a-2, let-7c, and let-7e) and group II (let-7a-1, let-7a-3, let-7b, let-7d, let-7f-1, let-7f-2, let-7g, let-7i, and miR-98) pre-let-7s in control and TUT7/4/2 knockdown (TUTKD) HeLa cells. A considerable portion of intact group II pre-let-7s was mono-uridylated (“Mono-U”) in control. Upon TUT7/4/2 knockdown, mono-uridylation of intact group II pre-let-7s substantially decreased. The reads whose length of trimming is > 0 were defined as “Trimmed”. The reads whose length of trimming is = 0 with none non-templated addition were defined as “Intact”. The reads whose length of trimming is = 0 with one non-templated uridine were defined as “Mono-U”. The rest reads were defined as “Other”. The percentage was calculated by normalizing with total read.

Dot plots of pre-let-7e (group I) and pre-let-7a-1 (group II). The status of 3′ trimming and 3′ U-tailing for each pre-miRNA read is represented by a circle on a two-dimensional matrix. The *x*-axis represents the length of 3′ trimming, and the *y*-axis represents the length of U-tail. Area of a circle is proportional to the relative abundance of the pre-miRNA reads. Reference sequence of the hairpin is shown below the dot plot. Position 0 indicates the 3′ end of genomic sequence of most abundant read in control HeLa cells. Pre-let-7e was rarely mono-uridylated in control and was not affected by TUT7/4/2 knockdown. In contrast, about half of pre-let-7a-1 was mono-uridylated in control, and the mono-uridylated reads almost disappeared upon TUT7/4/2 knockdown. The style of the figure was adopted from Zhao *et al* (2012).

Box plot of trimmed ratio and uridylation ratio of trimmed species of 54 pre-miRNAs, which have more than 400 reads. Dark red line indicates the median. Uridylation of trimmed pre-miRNAs decreased, while trimmed pre-miRNA reads increased upon TUT7/4/2 knockdown (TUTKD).

Dot plots and representative reads of pre-miR-26a-2. Representative reads are union of top 10 abundant reads in control or TUT7/4/2 knockdown (TUTKD) sample. Red letter, non-templated uridine; green letter, non-templated adenine; yellow box, non-templated tailing. Proportion (%) of each pre-miRNA species is indicated in each sample, and fold change was calculated by dividing the proportion of TUTKD (%) by the proportion of Control (%). Trimmed pre-miR-26a-2 reads were substantially uridylated. In TUT7/4/2 knockdown cells, trimmed pre-miRNA reads increased.

Knockdown of TUT7/4/2 resulted in a decrease in uridylation in the vast majority of pre-miRNAs, suggesting that TUT7/4/2 can uridylate most pre-miRNAs to some degrees (Fig4B and Supplementary Table S2). Adenylation was not affected significantly, confirming that TUT7/4/2 work mainly as uridylyl transferases on pre-miRNAs (Fig4B and Supplementary Table S2). Note that any terminal residue matching genomic sequence was considered as templated, so the modification rates are underestimated. To observe the effect of TUT7/4/2 knockdown, we first investigated mono-uridylation pattern of group I and group II pre-let-7s (Fig4C). Consistent with the previous study (Heo *et al*, 2012), a significant portion (36.2%) of group II pre-let-7s is mono-uridylated in control cells, while mono-uridylation decreased to 7.0% upon TUT7/4/2 knockdown. On the other hand, a proportion of group I pre-let-7s was rarely mono-uridylated (1.08%) in the control HeLa cells (Fig4C). Notably, trimmed pre-miRNAs accumulated more than 2-fold in both group I and group II pre-let-7s when TUT7/4/2 are depleted, suggesting that TUT7/4/2 may act to facilitate removal of trimmed pre-miRNAs.

To observe trimming and uridylation pattern of pre-let-7s in detail, we drew dot plots which show the fractions of pre-miRNAs that were trimmed of specific length (*x*-axis) and gained a U-tail of certain size (*y*-axis) (Fig4D). Although the majority of pre-let-7a-1 (group II) was mono-uridylated in control cells, uridylation reduced dramatically upon TUT7/4/2 knockdown (Fig4D, right). We also observed that shorter pre-let-7a-1 species increased upon TUT7/4/2 knockdown. Pre-let-7e (group I) was not strongly affected by TUT7/4/2, yet we detected a modest decrease in uridylation and an accumulation of trimmed pre-let-7e (Fig4D, left).

Next, we analyzed 54 pre-miRNAs that yielded sufficient reads for analysis (> 400 total reads), including pre-let-7s. For most pre-miRNAs, a substantial fraction of reads corresponded to the 3′ truncated fragments (Fig4E, left, 16.7% in control cells and 32.7% in TUT knockdown cells, median). A significant portion of the trimmed fragments was uridylated in control cells (Fig4E, right, 16.3%, median). For some pre-miRNAs (7 of 54 pre-miRNAs), more than 40% of trimmed reads were uridylated (Supplementary Table S3). When TUT7/4/2 were depleted, the uridylation frequency decreased to less than half (Fig4E, right, 6.3%, median). These results indicate that uridylation is not restricted to the let-7 family.

Figure4F presents pre-miR-26a-2 as an example. Nearly 33% of pre-miR-26a-2 reads were recessed from the 3′ end by 10–12 nt. U tails are found mostly on the recessed pre-miR-26a-2; about 70% of 10-nt trimmed reads carried an oligo-U tail in control cells. In TUT7/4/2-depleted cells, uridylation was reduced (from 70 to 24%) and, at the same time, shorter reads (11 or 12 nt trimmed) accumulated more than twice (12 nt, from 9.2 to 18.7%; 13 nt, from 13.2 to 35.5%). Many other pre-miRNAs including pre-miR-191 showed similar patterns to that of pre-miR-26a-2 (Supplementary Table S3). Thus, our results suggest that TUTases uridylate 3′ trimmed pre-miRNAs in general, which may lead to destabilization of defective pre-miRNAs. Of note, accumulation of trimmed pre-miRNAs upon TUT7/4/2 knockdown does not greatly affect the mature miRNA levels in general (Heo *et al*, 2012; Thornton *et al*, 2012; Liu *et al*, 2014) because 3′ trimmed pre-miRNAs are defective for Dicer processing.

## Discussion

**Figure 5:**
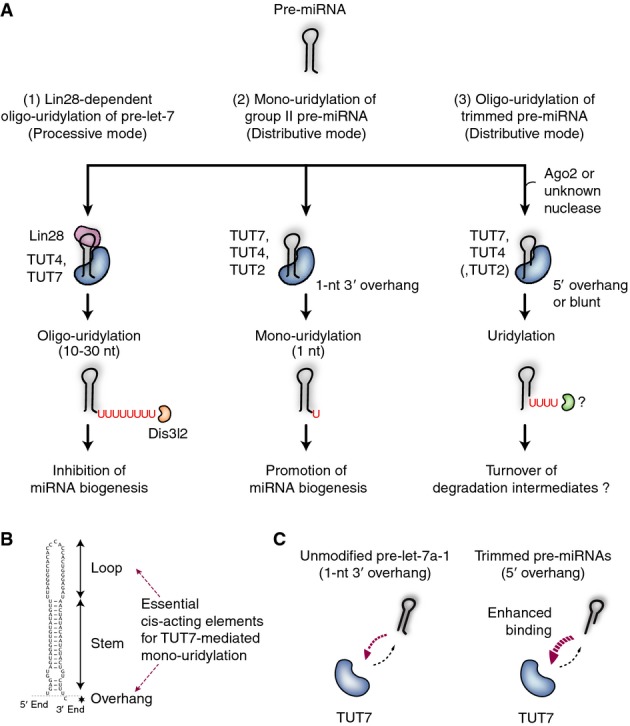
Models for pre-miRNA uridylation by TUT7

Our work demonstrates that there are three distinct pathways of pre-miRNA uridylation (Fig5A). (1) In embryonic cells and certain cancer cells, TUT4 (and TUT7, to a lesser extent) associates with Lin28 and oligo-uridylates pre-let-7 specifically. Lin28-mediated oligo-uridylation blocks pre-let-7 processing and promotes degradation by DIS3L2. (2) In the absence of Lin28, TUT7/4/2 mono-uridylate group II pre-miRNAs (with a 1-nt 3′ overhang), which include most of pre-let-7 members. Mono-uridylation of group II pre-miRNAs shapes an optimal 3′ end overhang for efficient processing. (3) In this study, we uncover another pathway in which oligo-U tails are added by TUT7/4 to truncated pre-miRNAs with a 5′ overhang. TUT2 seems to be less active than TUT7/4 (Fig2B and Supplementary Fig S2D). The oligo-U tails on the trimmed pre-miRNAs may promote rapid degradation of non-functional pre-miRNA species.

Pre-miRNA uridylation by TUT7/4/2 in microRNA biogenesis pathway. (1) In embryonic cells and certain cancer cells, Lin28 recruits TUT4 (and TUT7, to a lesser extent) to pre-let-7 for oligo-uridylation. This reaction is processive. Oligo-uridylated pre-let-7 cannot be processed to mature miRNA and is instead degraded by Dis3l2. (2) Group II pre-miRNA with a 1-nt 3′ overhang is mono-uridylated by TUT7/4/2, which creates an optimal end structure for Dicer-mediated processing. TUT7 plays a major role in HeLa cells but TUT4/2 also contribute to mono-uridylation. The reaction is distributive, and the interaction between TUT and pre-let-7 is infrequent. (3) Trimmed pre-miRNAs are uridylated by TUT7 and TUT4, which promote their degradation. TUT2 seems to be less active than TUT7/4. As the interaction between enzyme and substrate is frequent, multiple cycles of distributive uridylation result in oligo-uridylation.

*Cis*-acting elements of pre-let-7a-1 for mono-uridylation by TUT7. The overhang and the terminal loop are recognized by the C-terminal half of TUT7.

Model of interaction between TUT7 and its RNA substrates. For 3′ trimmed pre-miRNAs, binding rate to TUT7 increased, resulting in enhanced uridylation activity.

This study reveals the molecular mechanism of uridylation of group II pre-miRNA (Fig5). Our results indicate that TUT7 distinguishes pre-miRNA substrates at the binding step by recognizing the cis-acting elements. Pre-let-7a-1 with a 2-nt 3′ overhang is not efficiently uridylated due to infrequent binding of RNA to TUT7. Ablation of the terminal loop of pre-let-7a-1 also reduced the binding frequency, indicating that TUT7 recognizes both the overhang and the terminal loop. Given that TUT4/2 do not recognize the terminal loop (Supplementary Fig S2C), TUT7 may be used preferentially to uridylate pre-let-7s while TUT4/2 may have a broader specificity.

We have drawn the energy landscape that explains kinetics of group II pre-miRNA mono-uridylation (Supplementary Fig S6A and B). RNA binding is represented as a transition from a ‘free TUT7’ state to ‘RNA-bound TUT7’ (termed RNA+TUT7). The structural motif of RNA is probed by TUT7 at the transient state, (RNA+TUT7)*. The energy barrier (ΔG, then ΔΔG = − RT ln(k^variant^_on_/k^Unmodified^_on_)) between the ‘free TUT7’ state and the transient state becomes higher by 2.0 ± 0.5 or by 3.5 ± 0.1 (kJ/mol) if pre-let-7a-1 is mono-uridylated (pre-let-7a-1 +U) or if the terminal loop is removed from the RNA substrate (pre-let-7a-1 L4), respectively (Supplementary Fig S3B). In contrast, the energy barrier becomes lower when a pre-let-7a-1 variant contains a long 5′ overhang (ΔΔG(ΔCUUUC) = −2.7 ± 0.9 kJ/mol; and ΔΔG(Ac-pre) = −4.3 ± 0.6 kJ/mol) (Supplementary Fig S3B). However, the energy barrier between (RNA+TUT7) and (RNA+TUT7)* does not appear to change as significantly as the energy barrier of binding does. In summary, in the energy landscape of the TUT7-RNA interaction, it is the transient state at which TUT7 probes two cis-acting elements (the terminal loop and the overhang) and discriminates its RNA substrates.

Non-canonical overhang structures of pre-miRNAs (e.g. 1-nt 3′ overhang or 5′ overhang) increase uridylation efficiency. TUT7 oligo-uridylates 3′ trimmed pre-miRNAs at a much higher rate than unmodified pre-let-7a-1 due to enhanced binding (Figs2B, 3G and 5C). Intriguingly, unlike the processive reaction observed with Lin28-dependent oligo-uridylation of pre-let-7 (Yeom *et al*, 2011), oligo-uridylation of 3′ trimmed pre-miRNAs results from successive uridylation in a distributive mode (Fig5A and C). This explains why apparent ‘oligo’-uridylation is observed even for unmodified pre-let-7a-1 when a large amount of TUT enzyme is used or when reaction time is extended (Fig1D and Supplementary Fig S2). The distributive activity of human TUT7 is consistent with a recent structural study, which showed that Cid1, homolog of TUT7 in *S. pombe*, uridylates single-stranded RNA in a distributive manner (Yates *et al*, 2015).

Deep sequencing results suggest that TUT7 (and its paralogues) uridylates 3′ trimmed pre-miRNAs *in vivo* as well as *in vitro* and that the uridylation is likely to induce degradation of the 3′ trimmed pre-miRNAs. This result is consistent with a recent finding by Mourelatos and colleagues that defective Ago-bound pre-miRNAs are uridylated by TUT7/4 and degraded by exosome (DIS3 and RRP6) in mouse embryonic fibroblasts (Liu *et al*, 2014). They also reported that TUT7/4 associate with exosome, and this interaction may facilitate degradation of pre-miRNA. Our study confirms and expands the role of uridylation in removal of defective pre-miRNAs and further provides with mechanistic insights into the differential uridylation by TUTs. Moreover, our work explains their intrinsic preference for trimmed pre-miRNAs at the molecular level. Sensing the overhang structure, TUTs can employ multiple modes of action and thereby have versatile consequences of either repairing or removing pre-miRNAs depending on the molecular and cellular contexts.

## Materials and Methods

### Cell culture and transfection

HeLa and HEK293T (mycoplasma-free) cells were maintained in DMEM (Welgene) supplemented with 9% fetal bovine serum (Welgene). For RNAi, HeLa cells were transfected with 42 nM of siRNA by using Lipofectamine 2000 (Life Technologies). For simultaneous knockdown of three TUTs, equal amounts (14 nM) of siTUT7, siTUT4, and siTUT2 were combined. Transfection was performed two times over 4 days. For ectopic expression of proteins, HEK293T cells were transfected with plasmids by calcium phosphate method. The sequences of siRNA are listed in Supplementary Table S4.

### Mutagenesis of TUT7

To prepare TUT7 deletion mutants, PCR products of TUT7 deletion mutants were subcloned into FLAG-pCK vector for expression in human cells, at the BamHI and NotI sites. Primer sequences used for PCR are as follows. For ΔZF1, 5′-GGCATTGCCATTGACAAAGTGGTAC-3′ (forward) and 5′-TCATGATTCCTGCTGGGTCCTC-3′ (reverse) were used. For ΔNTr1, 5′-CCTGAAGAAGGAGGTCTGCCACC-3′ (forward) and 5′-TCATGATTCCTGCTGGGTCCTC-3′ (reverse) were used. For ΔPAP1, 5′-CACTTTACCCACTCAGTACAGGGCC-3′ (forward) and 5′-TCATGATTCCTGCTGGGTCCTC-3′ (reverse) were used. For NTr2-PAP2, 5′-CAGCTAGAACCTCTGCCACCATTAAC-3′ (forward) and 5′-GTCCTTTGGAAATCCCTTGACAGG-3′ (reverse) were used.

### Immunoprecipitation and *in vitro* uridylation

For immunoprecipitation of FLAG-TUTases, HEK293T cells grown on 15-cm dishes were collected 48 h after transfection of FLAG-TUTase expression plasmids. The cells were incubated with buffer D (200 mM KCl, 20 mM Tris [pH 8.0], 0.2 mM EDTA) containing protease inhibitor for 20 min followed by sonication on ice and centrifugation for 30 min at 4°C. The supernatant was incubated with 10 μl of anti-FLAG antibody-conjugated agarose beads (anti-FLAG M2 affinity gel, Sigma) with constant rotation for 2 h at 4°C. The beads were washed three times with buffer D. The reaction was performed in a total volume of 15 μl in 3.2 mM MgCl_2_, 1 mM DTT, 0.25 mM UTP, 20 U RNasin® Ribonuclease Inhibitor (Promega), 5′ end-labeled RNA of 0.2 nM, and 7.5 μl of immunopurified TUTases in buffer D. When uridylation assay was done with recombinant TUT7 (951–1,495 a.a), 6.7 nM of rTUT7 was used in Fig1D and Supplementary Fig S2A; 13.4 nM of rTUT7 was used in Supplementary Fig S2B. The reaction mixture was incubated at 37°C for 30 s−20 min. For *in vitro* uridylation assay with dilution experiment (Supplementary Fig S4C), 26.7 nM of recombinant TUT4 (rTUT4, 267–1,312 a.a) and 53.6 nM of recombinant Lin28b (rLin28b) were used. The reaction mixture was diluted four times with prewarmed reaction buffer after 20 seconds. The RNA was purified from the reaction mixture by phenol extraction and analyzed on 6% urea polyacrylamide sequencing gel with 7 M urea (20 × 40 cm, 0.4 mm thick). The gel was directly exposed to Phosphor Imaging Plate (Fujifilm) and was read with the Typhoon FLA 7,000. Unmodified pre-let-7a-1 and variants were synthesized by ST pharm. The pre-miRNAs were labeled at the 5′ end with T4 polynucleotide kinase (Takara) and (γ-^32^P) ATP. The sequences of pre-miRNAs are listed in Supplementary Table S4.

### Quantification of *in vitro* uridylation data

*In vitro* uridylation data are quantified as described in (Lim *et al*, 2014). The signal intensity profiles (20 pixels/mm) were calculated from the whole blot phosphor images using Fujifilm MultiGauge v3.0. For each lane, background signal is estimated using the arithmetic mean of the 25^th^ and the 50^th^ percentiles of the signal intensities. The signal intensities were subtracted by the estimated background level, then clipped to zero so that all intensities have zero or positive values. For the alignment of size marker bands, the signals from a marker lane were transformed to the first and second derivatives using Savitzky–Golay filter (window = 31 pixels, order = 3). The marker positions were detected by searching points where the sign of first derivative turns from positive to negative, and the second derivative is smaller than −100. The detected positions of marker bands were verified by visual inspection. The function between physical position in the gel and RNA size was defined using cubic spline interpolation. The density of RNA amount in size space was calculated using the first-order discrete differences of equal-width samples (0.1 nt) from cumulative density of the original intensity values. For the average length of extensions, the position having maximum signal intensity in the 0-min sample is used as a reference position. The average length of extension was derived from an equation, 

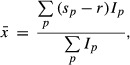
where 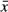 is the average length of extension, *p* is a position in the gel (by 0.1 nt-wide intervals), *s*_*p*_ is the RNA size in nucleotides count for position *p*, *r* is the reference size of unextended RNA, and *I*_*p*_ is signal intensity for position *p*. We excluded signals from degraded products (shorter than the reference size by 3 nt) in the calculation of average extension.

### Western blotting analysis

Proteins were resolved with 10% SDS–polyacrylamide gels and transferred to Immobilon-P transfer membrane (Millipore). Primary antibodies used were rabbit anti-FLAG (Sigma, F7425, 1:1,000).

### Purification of recombinant proteins

Recombinant TUT7 951–1,495 (rTUT7) and His-rTUT7 were prepared as previously described (Lim *et al*, 2014). For purification of recombinant TUT4 protein, human TUT4 267–1,312 was inserted into a self-modified pMAL expression vector, which fuses a hexa-His tag plus a maltose-binding protein tag at the N-terminus to the target protein. The plasmid was transformed into *E. coli* BL21(DE3)-RIL strain (Stratagene). The cells were cultured at 37°C until OD600 reached 1.0, and then the protein expression was induced with 1 mM IPTG at 16°C overnight. The hexa-His-MBP tagged protein was purified using a HisTrap FF column (GE Healthcare). The tag was cleaved by TEV protease and further removed by a second step HisTrap FF column (GE Healthcare) purification. The target protein was further purified by a Heparin FF column (GE Healthcare) and a Hiload Superdex G200 16/60 column (GE Healthcare). Recombinant Lin28b was prepared as previously described (Yeom *et al*, 2011).

### Sample preparation and RNA labeling for single-molecule measurements

RNA samples were labeled and prepared as previously described (Yeom *et al*, 2011).

### Single-molecule fluorescence microscopy

The fluorescent label Cy3 was imaged using prism-type total internal reflection microscopy, through excitation by a 532 nm (Compass 215M-50, Coherent). Cy5 was imaged using a 640 nm solid-state laser (CUBE 640-100C, Coherent). Fluorescence signals from single molecules were collected through a 60× water immersion objective (UPlanSApo, Olympus) with an inverted microscope (IX71, Olympus). Scattering of the 532-nm and 640-nm laser beams was blocked with a 488/532/635-nm notch filter (NF01-488/532/635, Chroma). Subsequently, signals of Cy3 and Cy5 were spatially split with a dichroic mirror (λ_cutoff_ = 645 nm, Chroma) and recorded with an electron multiplying CCD camera (iXon 897, Andor Technology).

### Slide preparation and single-molecule assays

To eliminate non-specific surface adsorption of proteins and nucleic acids to a quartz surface (Finkenbeiner), piranha-etched slides were PEGylated over two rounds of PEGylation as described previously (Chandradoss *et al*, 2014). After assembly of a microfluidic chamber, slides were incubated for 1 min with 20 μl streptavidin (0.1 mg/ml, S-888, Invitrogen) followed by a washing step with 100 μl of buffer A (12.5 mM Tris–HCl (pH 8.0, AM9855G, Ambion), 150 mM NaCl (AM9760G, Ambion), 1 mM DTT (D9779, Sigma)). Anti-6×-His tag antibodies were specifically immobilized through biotin–streptavidin linkage by incubating the chamber with 20 μl of 300 nM biotinylated anti-6×-His tag antibodies (ab27025, Abcam). After 5 min of incubation, remaining unbound anti-6×-His tag antibodies were flushed away with 100 μl buffer A. Next, 30 μl of 200 nM recombinant TUT7 951–1,495 fused to a 6×-His (His-rTUT7) was incubated on the slide, allowing the His-rTUT7 molecules to bind the surface-immobilized antibodies. After 5 min of incubation, unbound His-rTUT7 molecules were flushed away with 100 μl imaging buffer A (0.5× buffer A substituted with_,_ 0.1 mg/ml glucose oxidase (G2133, Sigma), 4 μg/ml catalase (10106810001, Roche), 1 mM Trolox ((±)-6-hydroxy-2,5,7,8-tetramethylchromane-2-carboxylic acid, 238813, Sigma) and 0.1 mM UTP (18333-013, Ambion). Next, 1 nM labeled RNA substrate(s) was/were introduced in the chamber while imaging at room temperature (23 ± 1°C) to monitor the interaction of TUT7 with RNA in real time.

The frequency measurement requires an accurate ratio between the concentrations of a sample of interest and a reference sample. To account for differences in concentration, we adsorbed RNA molecules to a positively charged surface as follows. KOH-etched quartz slides were coated with a layer of positively charged poly-L-lysine. After 5 min of incubation with 20 μl 0.01% poly-L-lysine (P4707, Sigma), the chamber was washed with 100 μl of buffer A. After washing, two fluorescently labeled RNA substrates (Unmodified-Cy3, Variant-Cy5) were introduced into the microfluidic chamber. After 5 min of incubation, the remaining unbound substrate was washed away with 100 μl of imaging buffer A and data were obtained from 20 fields of view. For each construct, this procedure was repeated with three individual dilutions on three different slides. Mean number of counts was used to correct the relative binding frequency for concentration.

### Single-molecule data acquisition and analysis

A series of CCD images were acquired with laboratory-made imaging software at a time resolution of 0.03–0.1 s. Fluorescence time traces were extracted with an algorithm written in IDL (ITT Visual Information Solutions) that picked fluorescence spots above a threshold with a defined Gaussian profile. The extracted time traces were analyzed using laboratory-made Matlab algorithms (MathWorks) that selectively picked anticorrelated traces above a defined threshold. These selected traces were further analyzed using a laboratory-made Matlab algorithm to extract dwell times and the number of binding events per trace. The relative binding frequency plots were generated by dividing the total number of binding events of each construct with the correction factor obtained from the poly-L-lysine experiment, after which the variant was normalized against the unmodified construct.

To measure the binding frequency, Cy3 molecules were simultaneously excited over an area of 50 × 50 μm^2^ with 16% of the full laser power of the (8 mW) green laser (532 nm) and red laser (640 nm), while the time resolution was set to 0.03 s. Under these imaging conditions, we obtained a high signal-to-noise ratio that facilitated the automated analysis. For dwell time measurements, Cy5 molecules were excited with 8% of the full laser power (4 mW) green laser (640 nm) to minimize photobleaching of the Cy5 dye during our observation time. Meanwhile, the time resolution was set between at 0.1 s to collect a large enough number of photons per time bin.

### Pre-miRNA library preparation

To prepare pre-miRNA cDNA library, total RNA was separated on 15% urea-PAGE and RNAs of 40–120 nt were gel-purified. Size-fractionated RNAs were ligated to 3′ adaptor by using T4 RNA ligase 2, truncated (NEB). The 3′ adaptor-ligated RNA was separated on 12.5% urea-PAGE and RNAs of 60–140 nt were gel-purified. Size-fractionated RNAs were reverse-transcribed with a RT primer that is complementary to the 3′ adaptor by using SuperScript III (Life Technologies), followed by two-step PCR amplification. cDNA was firstly amplified with the RT primer and miRNA-specific forward primers for 10 cycles and secondly amplified for 10 cycles (12 let-7 family primers) or 13 cycles (43 other miRNA primers) with Phusion DNA polymerase (NEB). The sequences of miRNA-specific primers are shown in Supplementary Table S1. The cDNA libraries were separated on 6% native polyacrylamide gel, and DNAs of 150–225 bp were gel-purified. The library was sequenced on Illumina MiSeq (110 × 59 paired end run) with 50% of the PhiX control library (Illumina, FC-110-3001). All adapters and primers are synthesized by IDT. Oligonucleotide sequences except for miRNA-specific forward primers are shown in Supplementary Table S4.

### Processing for pre-miRNA sequencing

We used miRBase release 21 (Kozomara & Griffiths-Jones, 2014) and the UCSC hg38 genome assembly for the reference sequences of human pre-miRNAs and flanking regions. To reduce misalignments near the ends of miRBase hairpin sequences, we extended the miRNA precursor spans by 10 bp to both ends. The sequences were extended and retrieved from the genome assembly using BEDTools (Quinlan & Hall, 2010) slop and getfasta commands. The cDNA library was sequenced for 110 cycles with the small RNA workflow in Illumina MiSeq. Sequences were processed using Cutadapt (Martin, 2011) to trim the 5′-most fifteen nucleotides and clip 3′ adapter sequences out. The sequence at the 5′ end was removed because they originate from PCR primers for specific enrichment of pre-miRNAs and often include significant number of mismatches to known pre-miRNA sequences. Short sequences (< 15 nt) after trimming and clipping, and sequences without a 3′ adapter part were removed from the further analyses. The remaining sequences were aligned to 10-bp-extended miRBase hairpins explained above using BLAT (Kent, 2002) with options “−noTrimA −tileSize=8 −stepSize=4−minIdentity=70 −out=pslx”. From the output alignments, the best alignments among multi-mapped reads were chosen by following criteria in order: maximum matched bases, minimum mismatch, minimum number of gaps in query of alignment, minimum number of gaps in target of alignment, minimum number of gapped bases in query of alignment and minimum number of gapped bases in target of alignment (preferred first). For the selected alignments, all unaligned bases in 3′ ends of local alignments were regarded as non-templated additions. We additionally “rescued” the non-templated additions, which were matched to the reference sequence by ambiguity in A/U-rich sites. First, the regions to be re-examined for the rescue were defined as all subsequent bases containing only A or U immediately starting from the 3′ end of a sequenced read (/[AU]+$/in the regular expression). Then, all subsequent bases including the first mismatched base in the re-examination region were rescued so as to be regarded as non-templated additions. The source codes, workflows implemented in Snakemake (Koster & Rahmann, 2012), and interactive notebooks in IPython Notebook (Perez & Granger, 2007) used for the analyses in this study are freely available from https://github.com/hyeshik/bskim-2015-pre-miRNA.

### Determination of length of trimming and length of U-tail

For each pre-miRNA, the most frequent 3′ end position of templated portions of reads in the control was considered as the “reference end position”. Six hairpins (hsa-mir-16-2, hsa-mir-100, hsa-mir-222, hsa-mir-320a, hsa-mir-1248, and hsa-mir-1291) whose reference end positions were offset by more than 3 nt from the 3′ end of the mature miRNA from 3′ arm of the hairpin defined in the miRBase were removed from the subsequent analyses to exclude artifacts from the statistics. The length of trimming of each read was calculated by subtracting the position of last templated base in the read from the reference end position (positive for “trimmed”, negative for “extended” reads). Length of U-tail was defined as number of U residues in the non-templated additions without any other kind of nucleotidyl additions.

### Accession numbers

Sequenced reads have been deposited in the NCBI Gene Expression Omnibus (GEO) database (accession number GSE64482).

## Supporting Information

Source Data for Figure S4

Review Process File
