# Supplementary material for: TUT7 controls the fate of precursor microRNAs by using three different uridylation mechanisms
Source: EMBO J. 2015 May 15;34(13):1801–15. doi: 10.15252/embj.201590931 (PMC4516432; doi:10.15252/embj.201590931)
Supplement: Supplementary file 1 [file embj0034-1801-sd1.pdf]

A Domain organization

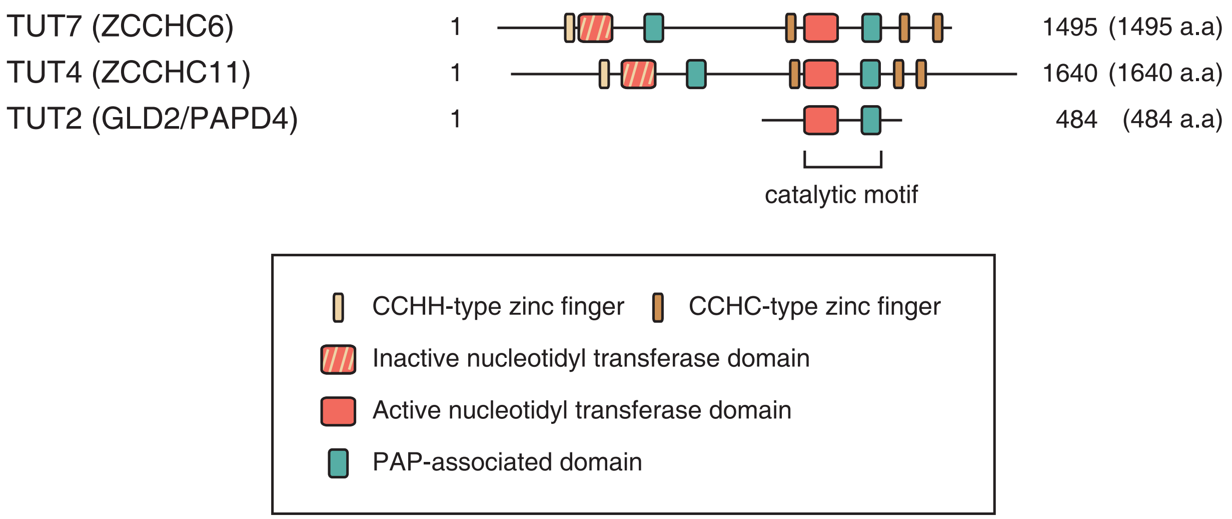

B Western blotting

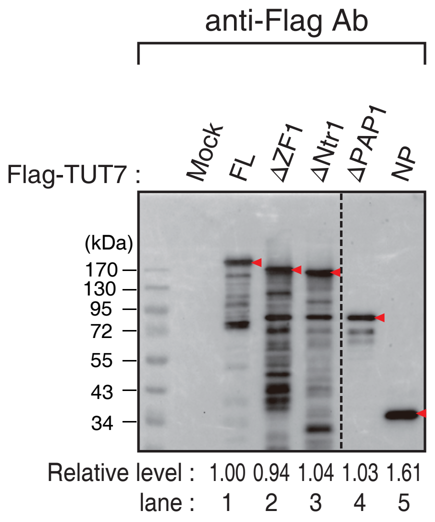

Supplementary Figure 1

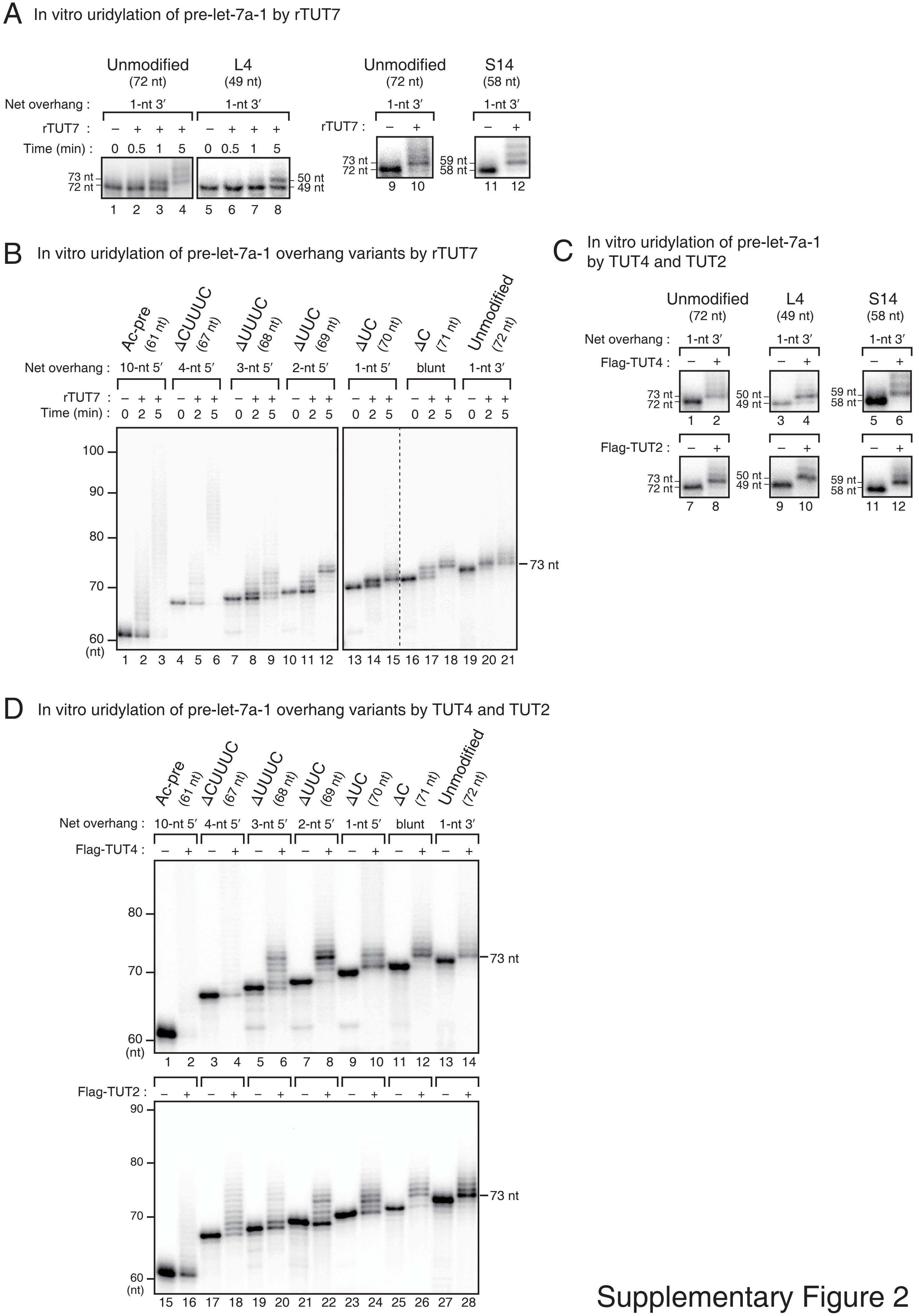

Supplementary Figure 2

A Dwell time analysis of unmodified pre-let-7a-1 and variants

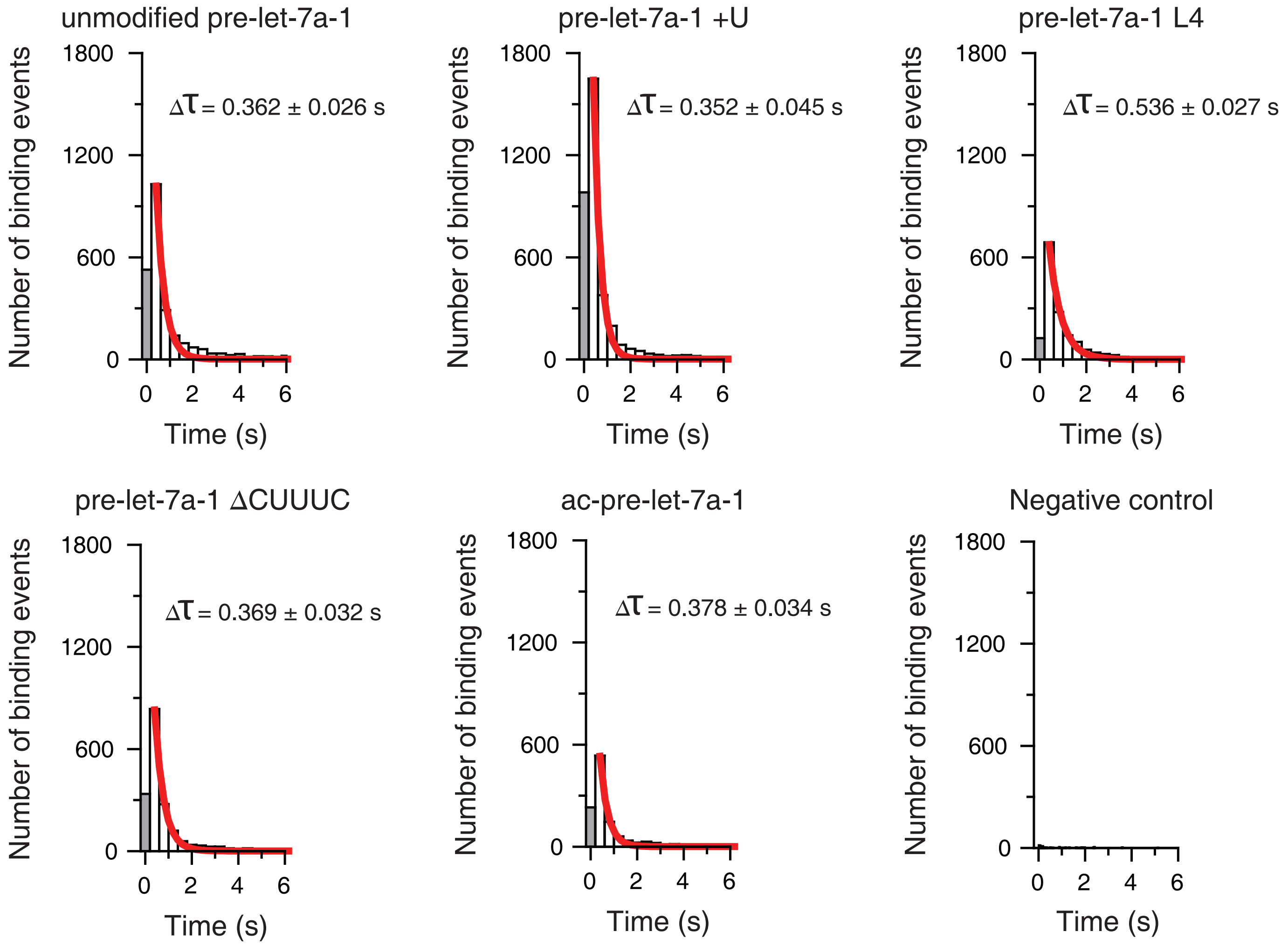

B Dissociation rate, binding rate and estimation of ΔΔG

	Unmodified	+U	L4	ΔCUUUC	Ac-pre
k_{off}	2.8 ± 0.2	2.9 ± 0.3	1.9 ± 0.1	2.7 ± 0.2	2.7 ± 0.2
$k_{on}^{Variant}/k_{on}^{Unmodified}$	1	0.4 ± 0.1	0.2 ± 0.0	3.3 ± 1.0	5.9 ± 1.0
ΔΔG (kJ/mol)	-	2.0 ± 0.5	3.5 ± 0.1	-2.7 ± 0.9	-4.3 ± 0.5

Supplementary Figure 3

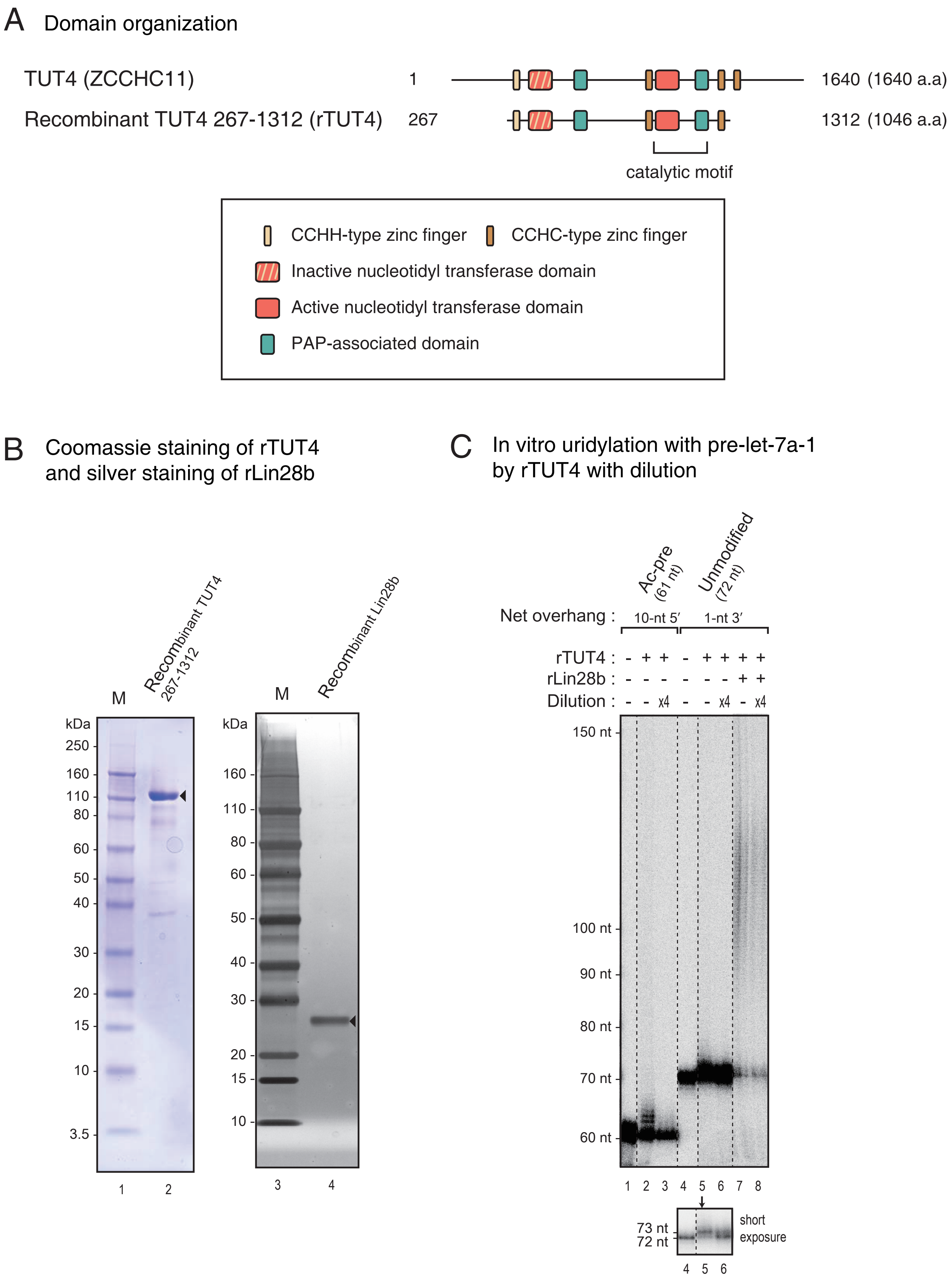

Supplementary Figure 4

Knockdown in HeLa cells

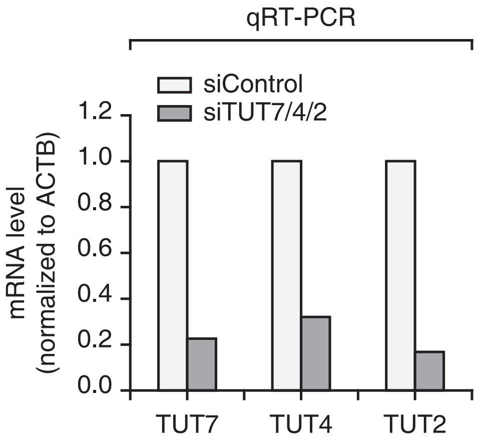

Supplementary Figure 5

A

Energy landscape of unmodified pre-let-7a-1, +U and L4

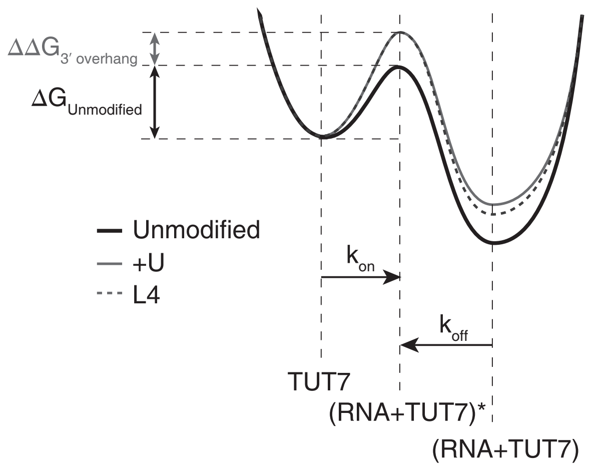

B

Energy landscape of unmodified pre-let-7a-1, ΔCUUUC and Ac-pre

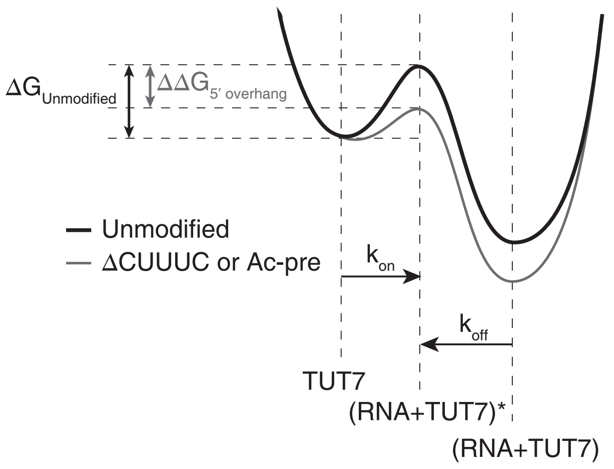

Supplementary Figure 6
