# Supplementary material for: TUT7 controls the fate of precursor microRNAs by using three different uridylation mechanisms
Source: EMBO J. 2015 May 15;34(13):1801–15. doi: 10.15252/embj.201590931 (PMC4516432; doi:10.15252/embj.201590931)
Supplement: Supplementary file 2 [file embj0034-1801-sd2.pdf]

Supplementary Table 1. Pre-miRNA deep sequencing primers

Primer name	Sequence (5′ to 3′)<br>5' Adapter + miRNA specific region
hsa-let-7a-1	GTTCAGAGTTCTACAGTCCGACGATCTGAGGTAGTAGGTTGTATAGTTTTAGG
hsa-let-7a-2	GTTCAGAGTTCTACAGTCCGACGATCTGAGGTAGTAGGTTGTATAGTTTAGAATT
hsa-let-7a-3	GTTCAGAGTTCTACAGTCCGACGATCTGAGGTAGTAGGTTGTATAGTTTGG
hsa-let-7b	GTTCAGAGTTCTACAGTCCGACGATCTGAGGTAGTAGGTTGTGTGGTT
hsa-let-7c	GTTCAGAGTTCTACAGTCCGACGATCTGAGGTAGTAGGTTGTATGGTTTAGA
hsa-let-7d	GTTCAGAGTTCTACAGTCCGACGATCAGAGGTAGTAGGTTGCATAGTTTTAG
has-let-7e	GTTCAGAGTTCTACAGTCCGACGATCAGGAGGTTGTATAGTTGAGGAGGAC
hsa-let-7f-1	GTTCAGAGTTCTACAGTCCGACGATCTGAGGTAGTAGATTGTATAGTTGTGG
hsa-let-7f-2	GTTCAGAGTTCTACAGTCCGACGATCTGAGGTAGTAGATTGTATAGTTTTAGGG
hsa-let-7g	GTTCAGAGTTCTACAGTCCGACGATCTGAGGTAGTAGTTTGTACAGTTTGAG
hsa-let-7i	GTTCAGAGTTCTACAGTCCGACGATCTGAGGTAGTAGTTTGTACAGTTTGAG
hsa-miR-98	GTTCAGAGTTCTACAGTCCGACGATCGGTAGTAAGTTGTATTGTTGTGGGGTAG
hsa-miR-100	GTTCAGAGTTCTACAGTCCGACGATCAACCCGTAGATCCGAACTT
hsa-miR-103a-1	GTTCAGAGTTCTACAGTCCGACGATCGGCTTCTTTACAGTGCTGC
hsa-mir-103a-2	GTTCAGAGTTCTACAGTCCGACGATCAGCTTCTTTACAGTGCTGCC
hsa-miR-105-1	GTTCAGAGTTCTACAGTCCGACGATCTCAAATGCTCAGACTCCTGT
hsa-miR-106b	GTTCAGAGTTCTACAGTCCGACGATCTAAAGTGCTGACAGTGCAGATAG
hsa-miR-148b	GTTCAGAGTTCTACAGTCCGACGATCAAGTTCTGTTATACACTCAGGCTG
hsa-miR-151a	GTTCAGAGTTCTACAGTCCGACGATCTCGAGGAGCTCACAGTCTAGTA
hsa-miR-182	GTTCAGAGTTCTACAGTCCGACGATCTTTGGCAATGGTAGAACTCA
hsa-mir-185	GTTCAGAGTTCTACAGTCCGACGATCTGGAGAGAAAGGCAGTTCC
hsa-miR-16-1	GTTCAGAGTTCTACAGTCCGACGATCTAGCAGCACGTAAATATTGGC
hsa-miR-191	GTTCAGAGTTCTACAGTCCGACGATCCAACGGAATCCCAAAAGC
hsa-miR-20a	GTTCAGAGTTCTACAGTCCGACGATCTAAAGTGCTTATAGTGCAGGTAGTG
hsa-miR-21	GTTCAGAGTTCTACAGTCCGACGATCTAGCTTATCAGACTGATGTTGACTG
hsa-miR-221	GTTCAGAGTTCTACAGTCCGACGATCACCTGGCATACAATGTAGATTTC
hsa-miR-222	GTTCAGAGTTCTACAGTCCGACGATCCTCAGTAGCCAGTGTAGATCCTG
hsa-miR-24-1	GTTCAGAGTTCTACAGTCCGACGATCTGCCTACTGAGCTGATATCAGT
hsa-miR-24-2	GTTCAGAGTTCTACAGTCCGACGATCTGCCTACTGAGCTGAAACAC
hsa-miR-26a-1	GTTCAGAGTTCTACAGTCCGACGATCTTCAAGTAATCCAGGATAGGCT
hsa-miR-27b	GTTCAGAGTTCTACAGTCCGACGATCAGAGCTTAGCTGATTGGTGAA
hsa-miR-30a	GTTCAGAGTTCTACAGTCCGACGATCTGTAAACATCCTCGACTGGA
hsa-miR-30c-1	GTTCAGAGTTCTACAGTCCGACGATCTGTAAACATCCTACACTCTCAGCT
hsa-miR-30d	GTTCAGAGTTCTACAGTCCGACGATCTGTAAACATCCCCGACTG
hsa-miR-320a	GTTCAGAGTTCTACAGTCCGACGATCGCCTTCTCTTCCCGGTT
hsa-miR-378a	GTTCAGAGTTCTACAGTCCGACGATCCTCCTGACTCCAGGTCCTG
hsa-miR-7-1	GTTCAGAGTTCTACAGTCCGACGATCTGGAAGACTAGTGATTTTGTTGTT
hsa-miR-93	GTTCAGAGTTCTACAGTCCGACGATCCAAAGTGCTGTTCGTGCA
hsa-miR-31	GTTCAGAGTTCTACAGTCCGACGATCAGGCAAGATGCTGGCATAGC
hsa-miR-101-1	GTTCAGAGTTCTACAGTCCGACGATCCAGTTATCACAGTGCTGATGCT
hsa-miR-345	GTTCAGAGTTCTACAGTCCGACGATCGCTGACTCCTAGTCCAGGGC
hsa-miR-9-2	GTTCAGAGTTCTACAGTCCGACGATCTCTTTGGTTATCTAGCTGTATGAGTG
hsa-miR-18a	GTTCAGAGTTCTACAGTCCGACGATCTAAGGTGCATCTAGTGCAGATAGT
hsa-miR-30b	GTTCAGAGTTCTACAGTCCGACGATCTGTAAACATCCTACACTCAGCTGT
hsa-miR-10b	GTTCAGAGTTCTACAGTCCGACGATCTACCCTGTAGAACCGAATTTGTG
hsa-miR-15a	GTTCAGAGTTCTACAGTCCGACGATCTAGCAGCACATAATGGTTTGTGG
hsa-miR-423	GTTCAGAGTTCTACAGTCCGACGATCTGAGGGGCAGAGAGCGA
hsa-miR-183	GTTCAGAGTTCTACAGTCCGACGATCTATGGCACTGGTAGAATTCACTGT
hsa-miR-196a-2	GTTCAGAGTTCTACAGTCCGACGATCTAGGTAGTTTCATGTTGTTGGGATT
hsa-miR-1226	GTTCAGAGTTCTACAGTCCGACGATCGTGAGGGCATGCAGGCC
hsa-miR-1248	GTTCAGAGTTCTACAGTCCGACGATCACCTTCTTGTATAAGCACTGTGC
hsa-miR-1291	GTTCAGAGTTCTACAGTCCGACGATCTGGCCCTGACTGAAGACCA
hsa-miR-1307	GTTCAGAGTTCTACAGTCCGACGATCTCGACCGGACCTCGACC
hsa-miR-148b	GTTCAGAGTTCTACAGTCCGACGATCAAGTTCTGTTATACACTCAGGCTG
hsa-miR-449b	GTTCAGAGTTCTACAGTCCGACGATCAGGCAGTGTATTGTTAGCTGGT

Supplementary Table 2. Uridylation ratio and adenylation ratio of pre-miRNAs

	Control					TUTKD (TUT7/4/2 KD)				
hairpin	Total reads	Uridylated reads	Uridylation ratio (%)	Adenylated reads	Adenylation ratio (%)	Total reads	Uridylated reads	Uridylation ratio (%)	Adenylated reads	Adenylation ratio (%)
hsa-mir-21	867159	7681	0.89	7010	0.81	666506	2783	0.42	4194	0.63
hsa-let-7f-1	834611	373627	44.77	7688	0.92	1783537	301817	16.92	10938	0.61
hsa-let-7b	238553	94091	39.44	2803	1.18	385241	69933	18.15	2923	0.76
hsa-let-7a-3	233797	63023	26.96	1674	0.72	375958	16043	4.27	1823	0.48
hsa-mir-423	230535	1807	0.78	328	0.14	120155	292	0.24	144	0.12
hsa-mir-93	191255	25939	13.56	3523	1.84	126946	11065	8.72	1625	1.28
hsa-let-7a-1	148857	68029	45.70	1162	0.78	473040	23603	4.99	2201	0.47
hsa-let-7d	143835	1777	1.24	1444	1.00	152561	1769	1.16	1662	1.09
hsa-mir-30a	106625	1533	1.44	136	0.13	93713	474	0.51	124	0.13
hsa-mir-30c-2	89507	100	0.11	485	0.54	74924	136	0.18	487	0.65
hsa-let-7e	74236	4853	6.54	314	0.42	39590	935	2.36	302	0.76
hsa-mir-182	69259	86	0.12	649	0.94	116461	77	0.07	1238	1.06
hsa-let-7g	67155	52143	77.65	1146	1.71	35234	13723	38.95	678	1.92
hsa-mir-98	57305	32109	56.03	573	1.00	79126	18401	23.26	955	1.21
hsa-mir-106b	55067	4982	9.05	625	1.13	115677	6217	5.37	2041	1.76
hsa-let-7f-2	52256	25562	48.92	439	0.84	164831	7639	4.63	868	0.53
hsa-mir-30c-1	43832	3477	7.93	495	1.13	37859	1590	4.20	303	0.80
hsa-mir-18a	39962	12405	31.04	201	0.50	97841	4873	4.98	1432	1.46
hsa-mir-20a	39085	2468	6.31	68	0.17	26602	584	2.20	57	0.21
hsa-mir-26a-2	36209	3787	10.46	883	2.44	62552	1199	1.92	1041	1.66
hsa-mir-27b	34227	13172	38.48	44	0.13	16735	1111	6.64	24	0.14
hsa-mir-191	30486	4651	15.26	778	2.55	32783	2159	6.59	688	2.10
hsa-mir-30d	30172	1445	4.79	300	0.99	15414	152	0.99	161	1.04
hsa-let-7a-2	28951	812	2.80	141	0.49	14769	234	1.58	107	0.72
hsa-mir-1226	26450	14292	54.03	8623	32.60	16448	6510	39.58	4353	26.47
hsa-let-7c	22036	1819	8.25	152	0.69	11607	375	3.23	95	0.82
hsa-mir-24-2	21298	448	2.10	98	0.46	15486	96	0.62	89	0.57
hsa-mir-15a	17914	12342	68.90	567	3.17	24921	3973	15.94	2696	10.82
hsa-mir-183	15719	2180	13.87	26	0.17	21571	1386	6.43	156	0.72
hsa-mir-196b	15147	4661	30.77	229	1.51	8656	640	7.39	281	3.25
hsa-mir-1307	14955	1536	10.27	197	1.32	10234	299	2.92	105	1.03
hsa-mir-17	8292	1918	23.13	7	0.08	6020	432	7.18	18	0.30
hsa-mir-148b	7756	5435	70.07	306	3.95	3639	1431	39.32	98	2.69
hsa-mir-26a-1	7629	511	6.70	48	0.63	8549	357	4.18	119	1.39
hsa-mir-24-1	5635	98	1.74	33	0.59	5284	21	0.40	36	0.68
hsa-let-7i	5118	2121	41.44	36	0.70	5933	557	9.39	78	1.31
hsa-mir-103a-1	4918	548	11.14	19	0.39	3158	93	2.94	26	0.82
hsa-mir-103a-2	4812	195	4.05	3	0.06	4472	25	0.56	6	0.13
hsa-mir-7-1	3759	594	15.80	7	0.19	7590	538	7.09	55	0.72
hsa-mir-16-1	3075	135	4.39	7	0.23	2194	36	1.64	7	0.32
hsa-mir-196a-2	3062	744	24.30	17	0.56	2111	317	15.02	17	0.81
hsa-mir-101-1	2778	22	0.79	7	0.25	2544	12	0.47	4	0.16
hsa-mir-185	2144	271	12.64	28	1.31	987	21	2.13	3	0.30
hsa-mir-15b	2025	4	0.20	10	0.49	2149	1	0.05	17	0.79
hsa-mir-105-1	1321	45	3.41	38	2.88	1116	15	1.34	35	3.14
hsa-mir-105-2	1229	34	2.77	34	2.77	867	8	0.92	46	5.31
hsa-mir-3607	1141	0	0.00	7	0.61	625	0	0.00	3	0.48
hsa-mir-30b	860	64	7.44	1	0.12	1263	41	3.25	7	0.55
hsa-mir-345	573	110	19.20	7	1.22	587	50	8.52	8	1.36
hsa-mir-221	527	236	44.78	2	0.38	731	113	15.46	3	0.41
hsa-mir-449a	506	7	1.38	0	0.00	425	1	0.24	0	0.00
hsa-mir-10b	496	15	3.02	0	0.00	332	9	2.71	4	1.20
hsa-mir-26b	459	18	3.92	18	3.92	351	4	1.14	9	2.56
hsa-mir-31	447	137	30.65	2	0.45	344	52	15.12	1	0.29
hsa-mir-10a	116	13	11.21	0	0.00	64	6	9.38	1	1.56
hsa-mir-34a	101	0	0.00	3	2.97	74	3	4.05	3	4.05
hsa-mir-449c	56	0	0.00	1	1.79	35	1	2.86	1	2.86
hsa-mir-107	55	13	23.64	0	0.00	50	0	0.00	0	0.00
hsa-mir-320e	46	3	6.52	1	2.17	29	2	6.90	0	0.00
hsa-mir-99a	41	0	0.00	0	0.00	42	0	0.00	1	2.38
hsa-mir-320c-1	29	8	27.59	4	13.79	31	3	9.68	1	3.23
hsa-mir-3653	26	0	0.00	0	0.00	2	0	0.00	0	0.00
hsa-mir-744	24	3	12.50	0	0.00	23	3	13.04	0	0.00
hsa-mir-6516	12	0	0.00	0	0.00	7	0	0.00	0	0.00
hsa-mir-30e	9	0	0.00	0	0.00	6	0	0.00	0	0.00
hsa-mir-4521	8	0	0.00	0	0.00	1	0	0.00	0	0.00
hsa-mir-18b	6	1	16.67	0	0.00	17	0	0.00	0	0.00
hsa-mir-4485	6	0	0.00	0	0.00	2	0	0.00	0	0.00
hsa-mir-1229	4	3	75.00	0	0.00	3	3	100.00	0	0.00
hsa-mir-664b	4	0	0.00	0	0.00	2	0	0.00	0	0.00
hsa-mir-195	3	0	0.00	0	0.00	4	0	0.00	0	0.00
hsa-mir-33b	3	0	0.00	0	0.00	4	0	0.00	0	0.00
hsa-mir-449b	3	0	0.00	0	0.00	2	0	0.00	0	0.00
hsa-mir-339	2	0	0.00	0	0.00	5	1	20.00	0	0.00
hsa-mir-6723	2	0	0.00	0	0.00	3	0	0.00	0	0.00
hsa-mir-9-1	2	0	0.00	0	0.00	4	0	0.00	0	0.00
hsa-mir-126	1	0	0.00	0	0.00	1	0	0.00	0	0.00
hsa-mir-6724-4	1	0	0.00	0	0.00	3	0	0.00	0	0.00

Supplementary Table 3. Trimming and uridylation of pre-miRNAs.

	Control					TUTKD (TUT7/4/2 KD)				
hairpin	Total reads	Trimmed reads	Trimmed %	Uridylated trimmed reads	Uridylated trimmed % (/trimmed reads)	Total reads	Trimmed reads	Trimmed %	Uridylated trimmed reads	Uridylated trimmed % (/trimmed reads)
hsa-mir-21	867159	9638	1.11	3766	39.07	666506	11043	1.66	2179	19.73
hsa-let-7f-1	834611	226588	27.15	67252	29.68	1783537	1385900	77.71	241235	17.41
hsa-let-7b	238553	99129	41.55	31917	32.20	385241	298189	77.40	61390	20.59
hsa-let-7a-3	233797	81163	34.72	2681	3.30	375958	307254	81.73	4592	1.49
hsa-mir-423	230535	2963	1.29	300	10.12	120155	1897	1.58	109	5.75
hsa-mir-93	191255	76663	40.08	24307	31.71	126946	58714	46.25	10929	18.61
hsa-let-7a-1	148857	66890	44.94	1805	2.70	473040	439060	92.82	7856	1.79
hsa-let-7d	143835	12074	8.39	577	4.78	152561	74402	48.77	1691	2.27
hsa-mir-30a	106625	2517	2.36	1337	53.12	93713	3129	3.34	470	15.02
hsa-mir-30c-2	89507	4654	5.20	93	2.00	74924	9239	12.33	136	1.47
hsa-let-7e	74236	5509	7.42	2447	44.42	39590	9182	23.19	721	7.85
hsa-mir-182	69259	1030	1.49	54	5.24	116461	2053	1.76	74	3.60
hsa-let-7g	67155	2565	3.82	243	9.47	35234	15450	43.85	1719	11.13
hsa-mir-98	57305	8678	15.14	2116	24.38	79126	53334	67.40	10266	19.25
hsa-mir-106b	55067	34440	62.54	1079	3.13	115677	104689	90.50	4693	4.48
hsa-let-7f-2	52256	22328	42.73	504	2.26	164831	152765	92.68	1821	1.19
hsa-mir-30c-1	43832	9302	21.22	3477	37.38	37859	17996	47.53	1590	8.84
hsa-mir-18a	39962	12830	32.11	735	5.73	97841	84185	86.04	2334	2.77
hsa-mir-20a	39085	11917	30.49	1996	16.75	26602	9240	34.73	542	5.87
hsa-mir-26a-2	36209	19666	54.31	3787	19.26	62552	45143	72.17	1199	2.66
hsa-mir-27b	34227	381	1.11	73	19.16	16735	311	1.86	33	10.61
hsa-mir-191	30486	14521	47.63	4648	32.01	32783	24502	74.74	2159	8.81
hsa-mir-30d	30172	2359	7.82	228	9.67	15414	3807	24.70	54	1.42
hsa-let-7a-2	28951	2291	7.91	809	35.31	14769	2690	18.21	234	8.70
hsa-mir-1226	26450	1883	7.12	700	37.17	16448	3752	22.81	729	19.43
hsa-let-7c	22036	3356	15.23	1811	53.96	11607	3337	28.75	375	11.24
hsa-mir-24-2	21298	2691	12.63	63	2.34	15486	2599	16.78	30	1.15
hsa-mir-15a	17914	4504	25.14	306	6.79	24921	21157	84.90	1351	6.39
hsa-mir-183	15719	1055	6.71	443	41.99	21571	4565	21.16	1067	23.37
hsa-mir-196b	15147	1824	12.04	334	18.31	8656	2830	32.69	134	4.73
hsa-mir-1307	14955	2710	18.12	423	15.61	10234	3139	30.67	198	6.31
hsa-mir-17	8292	886	10.68	177	19.98	6020	1903	31.61	198	10.40
hsa-mir-148b	7756	428	5.52	1	0.23	3639	1029	28.28	10	0.97
hsa-mir-26a-1	7629	1995	26.15	497	24.91	8549	3853	45.07	355	9.21
hsa-mir-24-1	5635	1302	23.11	59	4.53	5284	1466	27.74	8	0.55
hsa-let-7i	5118	424	8.28	19	4.48	5933	2444	41.19	218	8.92
hsa-mir-103a-1	4918	1313	26.70	233	17.75	3158	1033	32.71	64	6.20
hsa-mir-103a-2	4812	901	18.72	71	7.88	4472	1351	30.21	8	0.59
hsa-mir-7-1	3759	2149	57.17	591	27.50	7590	6698	88.25	538	8.03
hsa-mir-16-1	3075	394	12.81	77	19.54	2194	417	19.01	30	7.19
hsa-mir-196a-2	3062	1745	56.99	744	42.64	2111	1346	63.76	317	23.55
hsa-mir-101-1	2778	579	20.84	18	3.11	2544	982	38.60	10	1.02
hsa-mir-185	2144	33	1.54	11	33.33	987	34	3.44	2	5.88
hsa-mir-15b	2025	30	1.48	3	10.00	2149	77	3.58	1	1.30
hsa-mir-105-1	1321	150	11.36	23	15.33	1116	232	20.79	13	5.60
hsa-mir-105-2	1229	146	11.88	23	15.75	867	167	19.26	7	4.19
hsa-mir-3607	1141	16	1.40	0	0.00	625	6	0.96	0	0.00
hsa-mir-30b	860	388	45.12	8	2.06	1263	920	72.84	31	3.37
hsa-mir-345	573	107	18.67	5	4.67	587	305	51.96	31	10.16
hsa-mir-221	527	277	52.56	70	25.27	731	648	88.65	96	14.81
hsa-mir-449a	506	7	1.38	7	100.00	425	1	0.24	1	100.00
hsa-mir-10b	496	274	55.24	15	5.47	332	209	62.95	9	4.31
hsa-mir-26b	459	120	26.14	1	0.83	351	92	26.21	3	3.26
hsa-mir-31	447	229	51.23	137	59.83	344	185	53.78	52	28.11

Supplementary Table 4. List of oligonucleotides used in this study.

Oligonucleotide name	Sequence (5′ to 3′)
siCont	ACGAAAUUGGUGGCGUAGGTT
siTUT2_1	UUAAUCACCAGCACUAACGTT
siTUT2_2	AUUACAUGGAGCUUGAUGUTT
siTUT2_3	UAAAUCACCAUCACUGCUCTT
siTUT2_4	UUGAUCUCAGUUUCUGUUGTT
siTUT4_1	UAUAAAGUCUGAAGCAACCTT
siTUT4_2	UCUUUCUCUUCUUCAUUCCTT
siTUT4_3	UUUCUUAUGUCGUUUCUCCTT
siTUT4_4	AAUUUAAGCAGCUCUAACCTT
siTUT7_1	UUUUCUUGUGCCUCUUUUCTT
siTUT7_2	AUUUCUUUGUCCUCUUUGCTT
siTUT7_3	UUUGACACGAAUACUUAUCTT
siTUT7_4	UAAAUAGGUACUCAUGUUCTT
pre-let-7a-1 unmodified	UGAGGUAGUAGGUUGUAUAGUUUUAGGGUCACACCCACCACUGGGAGAUAACUAUACAAUCUACUGUCUUUC
pre-let-7a-1 +U	UGAGGUAGUAGGUUGUAUAGUUUUAGGGUCACACCCACCACUGGGAGAUAACUAUACAAUCUACUGUCUUUCU
pre-let-7a-1 L4	UGAGGUAGUAGGUUGUAUAGUUUUAUAACUAUACAAUCUACUGUCUUUC
pre-let-7a-1 S14	UGAGGUAGUAGGUUGUUAGGGUCACACCCACCACUGGGAGAUCAAUCUACUGUCUUUC
pre-let-7a-1 ΔC	UGAGGUAGUAGGUUGUAUAGUUUUAGGGUCACACCCACCACUGGGAGAUAACUAUACAAUCUACUGUCUUU
pre-let-7a-1 ΔUC	UGAGGUAGUAGGUUGUAUAGUUUUAGGGUCACACCCACCACUGGGAGAUAACUAUACAAUCUACUGUCUU
pre-let-7a-1 ΔUUC	UGAGGUAGUAGGUUGUAUAGUUUUAGGGUCACACCCACCACUGGGAGAUAACUAUACAAUCUACUGUCU
pre-let-7a-1 ΔUUUC	UGAGGUAGUAGGUUGUAUAGUUUUAGGGUCACACCCACCACUGGGAGAUAACUAUACAAUCUACUGUC
pre-let-7a-1 ΔCUUUC	UGAGGUAGUAGGUUGUAUAGUUUUAGGGUCACACCCACCACUGGGAGAUAACUAUACAAUCUACUGU
Ac-pre-let-7a-1	UGAGGUAGUAGGUUGUAUAGUUUUAGGGUCACACCCACCACUGGGAGAUAACUAUACAAUC
3' adapter	TGGAATTCTCGGGTGCCAAGG
RT primer	CAAGCAGAAGACGGCATACGA
2nd PCR Forward primer	AATGATACGGCGACCACCGAGATCTACACGTTCAGAGTTCTACAGTCCGA
2nd PCR Reverse primer	CAAGCAGAAGACGGCATACGAGATCGTGATGTGACTGGAGTTCCTTGGCACCCGAGAATTCCA
