# Supplementary material for: TUT7 controls the fate of precursor microRNAs by using three different uridylation mechanisms
Source: EMBO J. 2015 May 15;34(13):1801–15. doi: 10.15252/embj.201590931 (PMC4516432; doi:10.15252/embj.201590931)
Supplement: Supplementary file 3 [file embj0034-1801-sd3.docx]

**SUPPLEMENTARY FIGURE LEGENDS**

**Supplementary Figure S1. Domain organization of TUT7/4/2 and expression of TUT7 deletion mutants (related to Fig 1).**

(A) Domain organization of human TUT7, TUT4, and TUT2. Yellow, CCHH-type zinc finger; hatched red, inactive nucleotidyl transferase domain due to a sequence variation; green, PAP-associated domain; orange, CCHC-type zinc finger; red, nucleotidyl transferase domain. (B) Western blotting of immunoprecipitated TUT7 full-length (FL) and deletion mutants (ΔZF1, ΔNtr1, ΔPAP1, and NP). Each protein is indicated by red arrowheads. Dashed line indicates discontinuous lanes from the same gel.

**Supplementary Figure S2. In vitro uridylation of pre-let-7a-1 mutants by recombinant TUT7 951-1495, TUT4, and TUT2 (related to Fig 2).**

(A) In vitro uridylation of unmodified pre-let-7a-1, terminal loop mutant (L4), and stem mutant (S14) by recombinant TUT7 951-1495 (rTUT7) (4 min reaction for lanes 9 - 12). rTUT7 uridylated L4 mutant less efficiently than unmodified pre-let-7a-1 but uridylated S14 mutants as efficiently as unmodified pre-let-7a-1. (B) In vitro uridylation of pre-let-7a-1 overhang variants by rTUT7. 13.4 nM of rTUT7 was used. rTUT7 showed the same substrate preference as full-length TUT7. Dashed line indicates discontinuous lanes from the same gel. (C) In vitro uridylation of unmodified pre-let-7a-1, terminal loop mutant (L4), and stem mutant (S14) by immunopurified full-length TUT4 and immunopurified full-length TUT2 (20 min reaction for TUT4 and 15 min reaction for TUT2). TUT4 and TUT2, unlike TUT7, uridylated both L4 and S14 mutants efficiently. (D) In vitro uridylation of pre-let-7a-1 overhang variants by immunopurified full-length TUT4 and immunopurified full-length TUT2 (20 min reaction for TUT4 and 10 min reaction for TUT2). By and large, TUT7/4/2 showed similar substrate specificity to overhang variants. Unlike TUT7 and TUT4, TUT2 did not show enhanced uridylation to the pre-miRNAs with long 5′ overhangs (ΔCUUUC and Ac-pre).

**Supplementary Figure S3. Single-molecule assay of unmodified pre-let-7a-1 and variants (related to Fig 3).**

(A) Representative dwell time distributions of unmodified pre-let-7a-1 and variants fitted with a single-exponential decay curve. As all RNA substrates followed single-exponential decay, dissociation of the RNA substrate from TUT7 is a single-step process. The first data point (grey) was not included in the fit due to limited time resolution. Δτ represents average dwell time (n=3) ± standard error. Negative control was performed with unmodified pre-let-7a-1 without recombinant TUT7 protein immobilized. (B) Table of dissociation rate (k_off_), binding rate (k^Variant^_on_/k^Unmodified^_on_) and estimation of ΔΔG of unmodified pre-let-7a-1 and mutants.

**Supplementary Figure S4. In vitro uridylation of pre-let-7a-1 unmodified and ac-pre-let-7a-1 by rTUT4 (related to Fig 3).**

(A) Domain organization of recombinant protein of human TUT4 267-1312. Yellow, CCHH-type zinc finger; hatched red, inactive nucleotidyl transferase domain due to a sequence variation; green, PAP-associated domain; orange, CCHC-type zinc finger; red, nucleotidyl transferase domain. (B) Coomassie staining of recombinant TUT4 267-1312 resolved on NuPAGE® Bis-Tris gel and silver staining of recombinant Lin28b resolved on Bolt® 4-12% Bis-Tris Plus Gel. Each protein is indicated by arrowheads. M, size marker. (C) In vitro uridylation assay of ac-pre-let-7a-1 and unmodified pre-let-7a-1 by rTUT4 267-1312 with or without rLin28b. Reaction mixture was either not diluted or diluted after 20 seconds. While processive oligo-uridylation by rTUT4 and rLin28b was not inhibited by dilution, oligo-uridylation of ac-pre-let-7a-1 and mono-uridylation of unmodified pre-let-7a-1 were repressed by dilution, indicating that TUT4 is distributive enzyme. Dashed line indicates discontinuous lanes from the same gel. For over-exposed bands, image with short exposure is presented below.

**Supplementary Figure S5. TUT7/4/2 knockdown in HeLa cells for pre-miRNA deep sequencing (related to Fig 4).**

The mRNA levels of TUT7, TUT4, and TUT2 were measured by qRT-PCR with sequencing samples. The mRNA of all the three TUTs decreased to about 20% upon TUT7/4/2 knockdown.

**Supplementary Figure S6. Energy landscape of unmodified pre-let-7a-1 and variants (related to Fig 5).**

(A,B) Energy landscape of pre-let-7a-1 +U, and L4 (A) and ΔCUUUC, and Ac-pre (B). (RNA+TUT7)* indicates transient state of interaction between TUT7 and RNA substrate. When pre-let-7a-1 is mono-uridylated or when the terminal loop is diminished, the energy barrier increased. On the contrary, when the 3′ end of pre-let-7a-1 is shortened to have 5′ overhang, the energy barrier decreased. TUT7 distinguishes its RNA substrates at transient state.

**SUPPLEMENTARY TABLES LEGENDS**

**Supplementary Table S1. Pre-miRNA deep sequencing primers (related to Fig 4).**

Forward PCR primers used in the 1st PCR step of pre-miRNA deep sequencing. Primer consists of 5′ adapter and miRNA specific region.

**Supplementary Table S2. Uridylation ratio and adenylation ratio of pre-miRNAs (related to Fig 4).**

**Supplementary Table S3. Trimming and uridylation of pre-miRNAs (related to Fig 4).**

**Supplementary Table S4. List of oligonucleotides used in this study.**
