# Supplementary figures and images for: TUT7 controls the fate of precursor microRNAs by using three different uridylation mechanisms
Source: EMBO J. 2015 May 15;34(13):1801–15. doi: 10.15252/embj.201590931 (PMC4516432; doi:10.15252/embj.201590931)

B

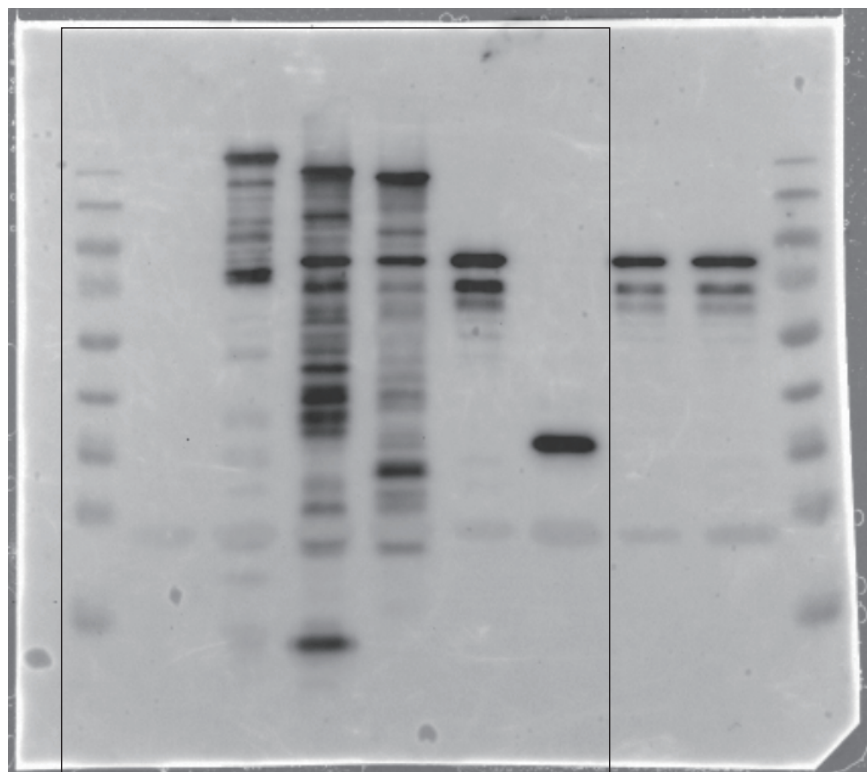

Supplement: Supplementary file 4 [file embj0034-1801-sd4.pdf]

A

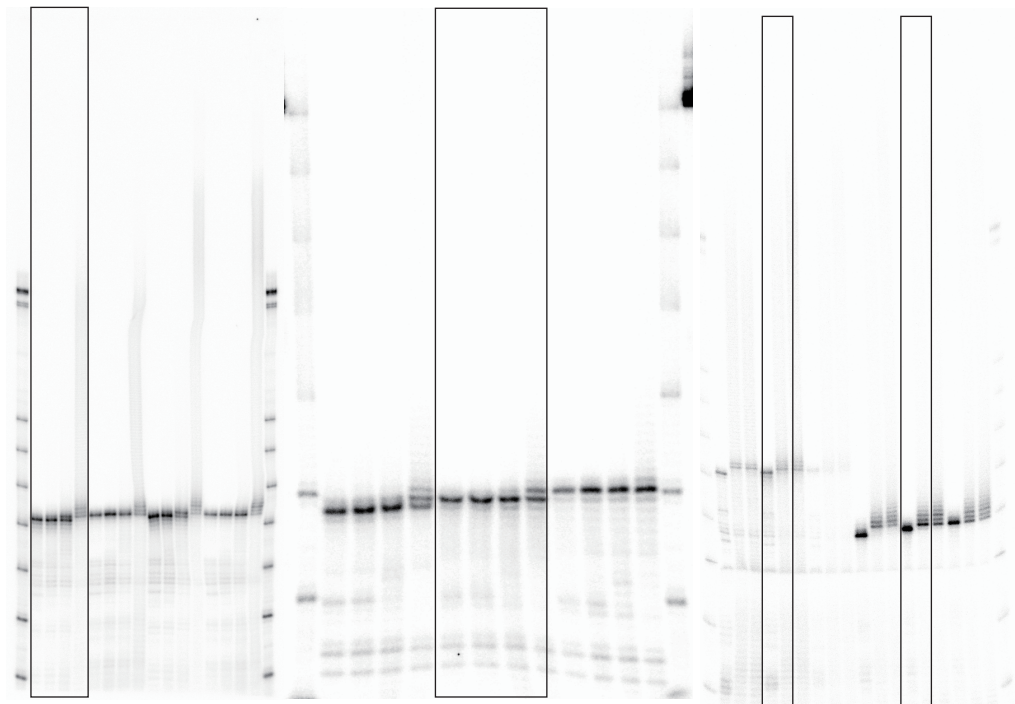

B

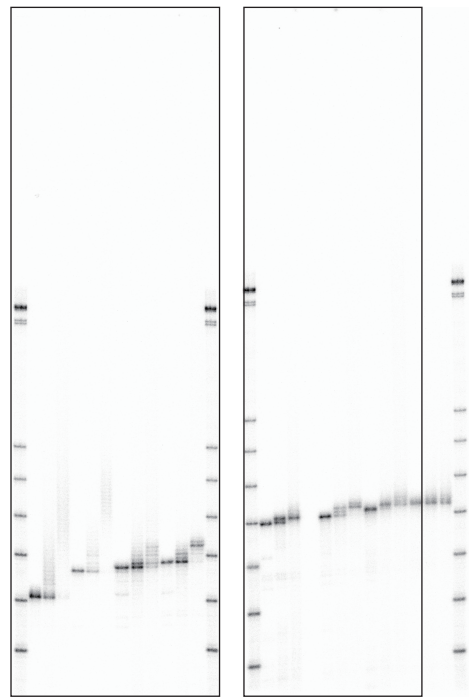

C

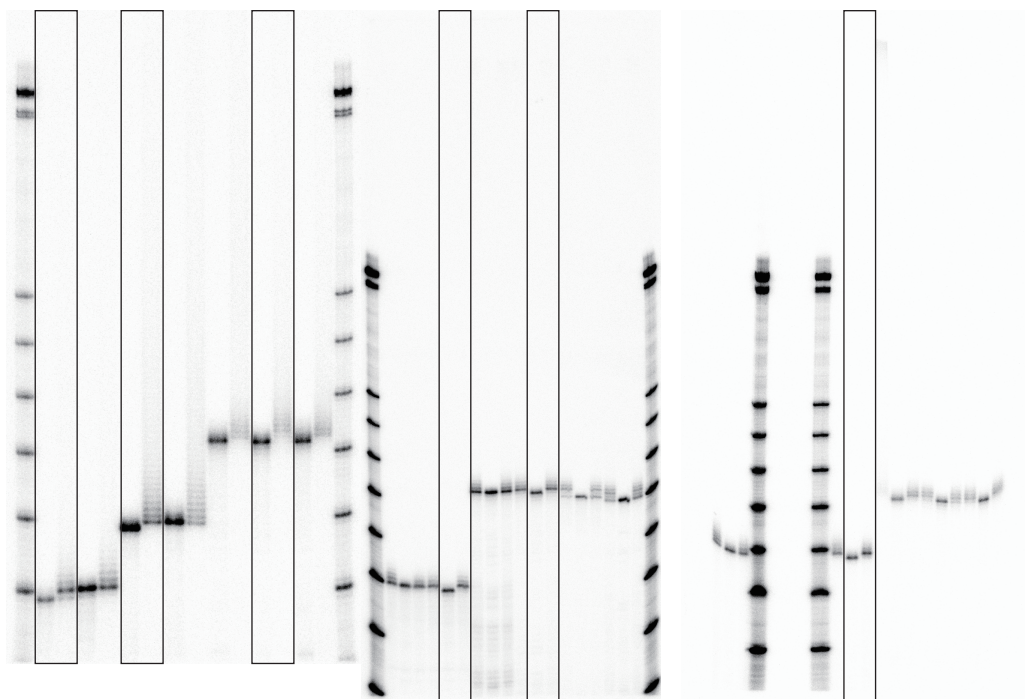

D

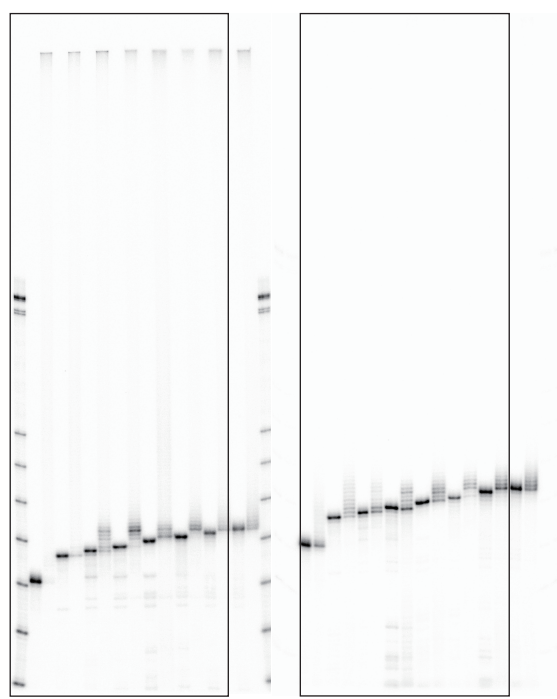

Supplement: Supplementary file 5 [file embj0034-1801-sd5.pdf]

B

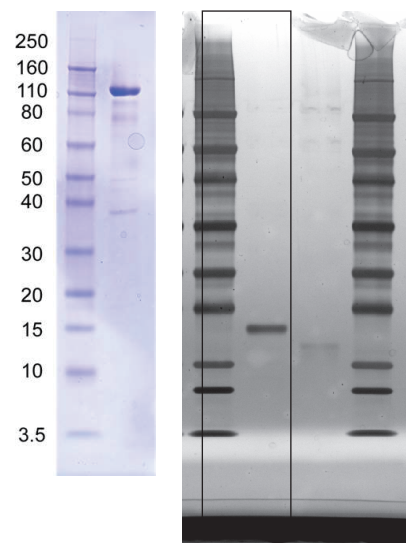

C

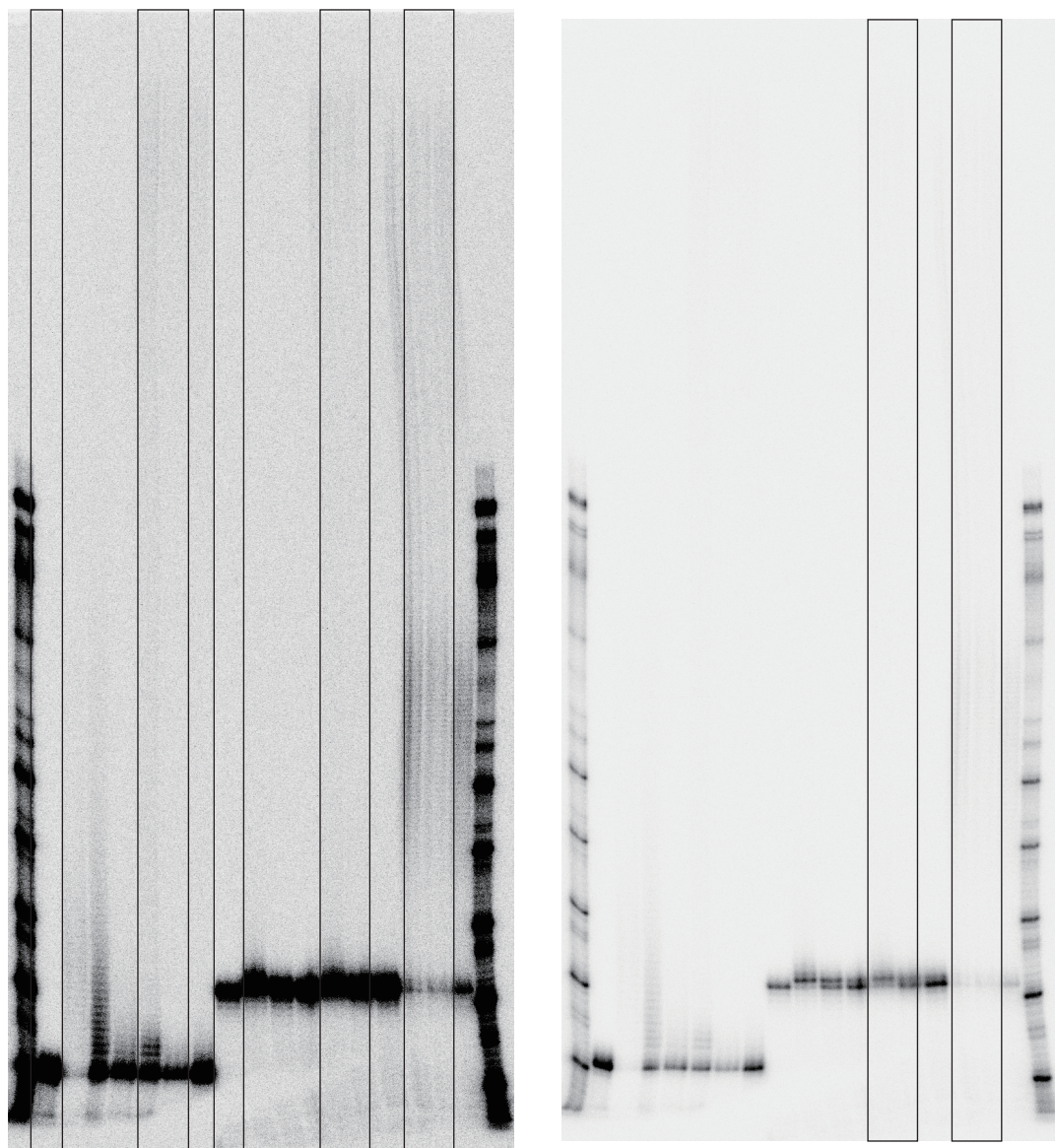

Supplement: Supplementary file 6 [file embj0034-1801-sd6.pdf]

B

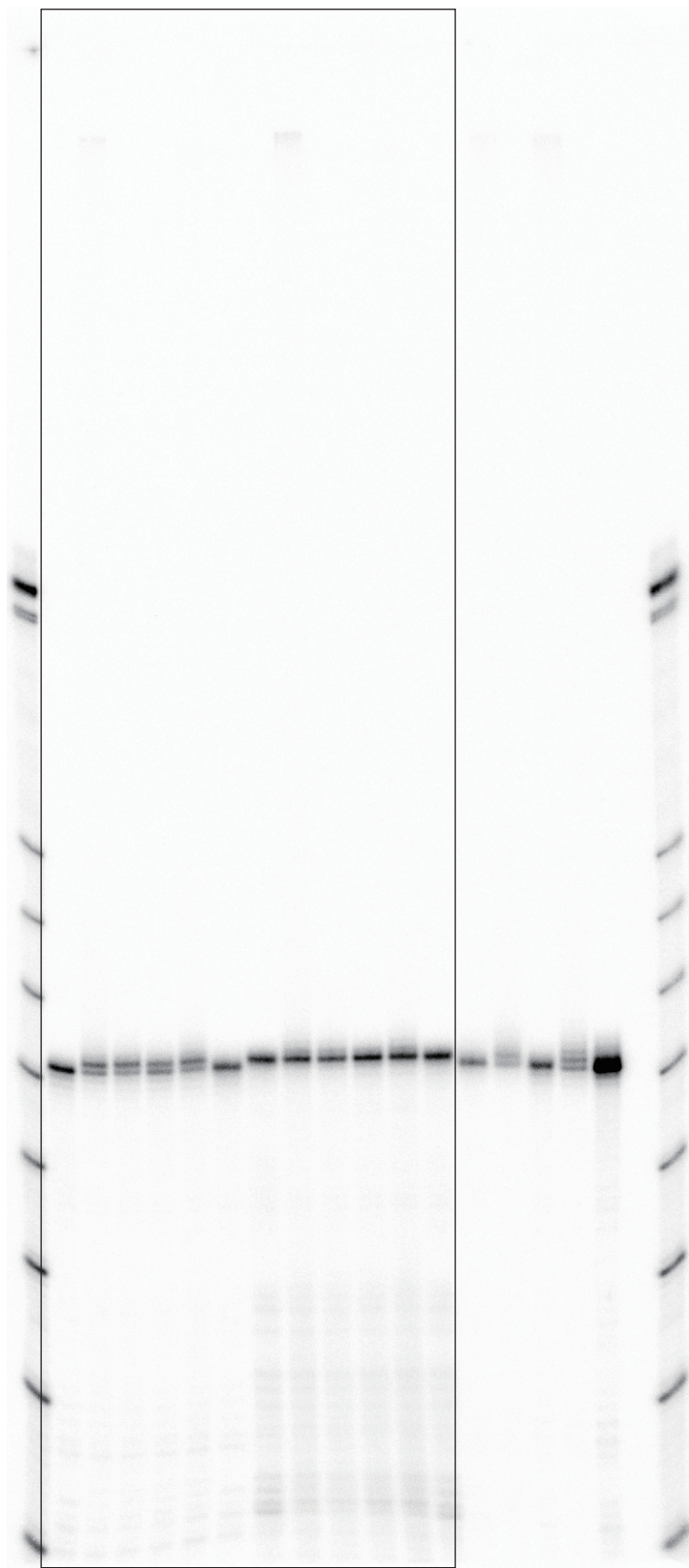

D

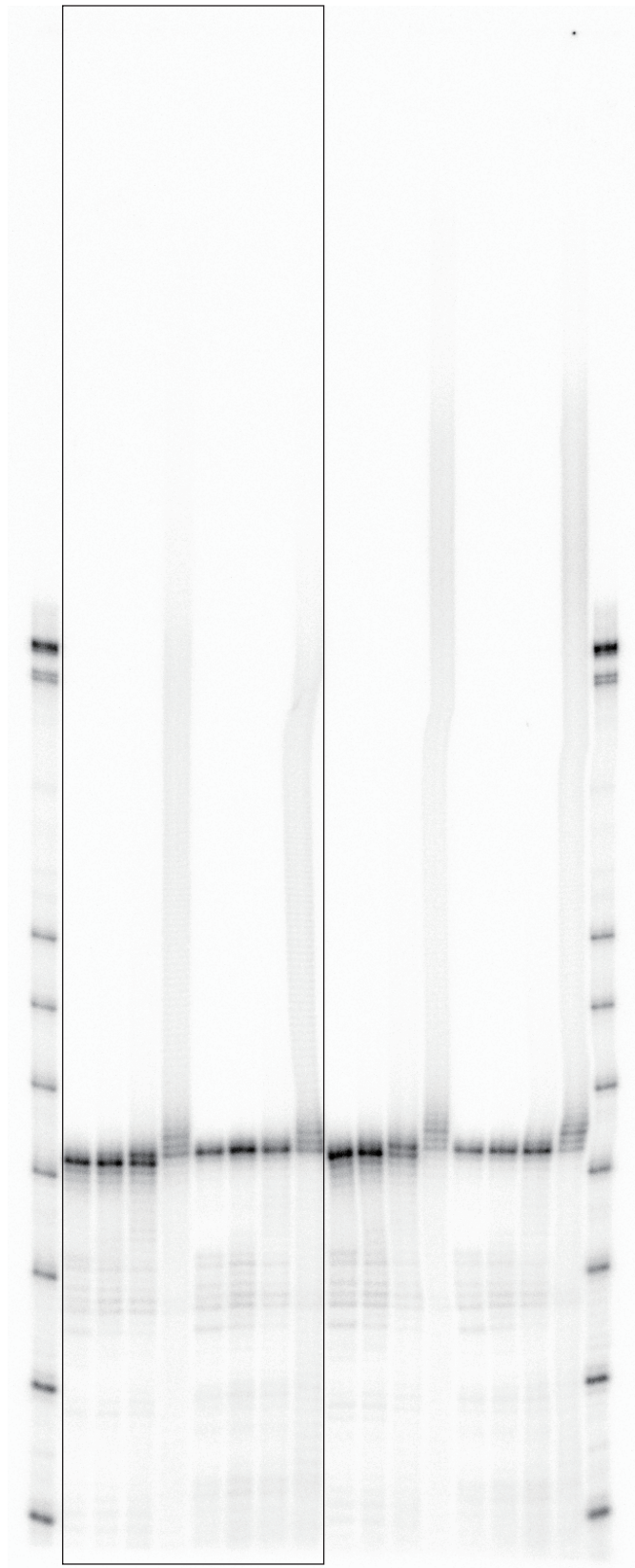

Figure 1 Kim et al

Supplement: Supplementary file 8 [file embj0034-1801-sd8.pdf]

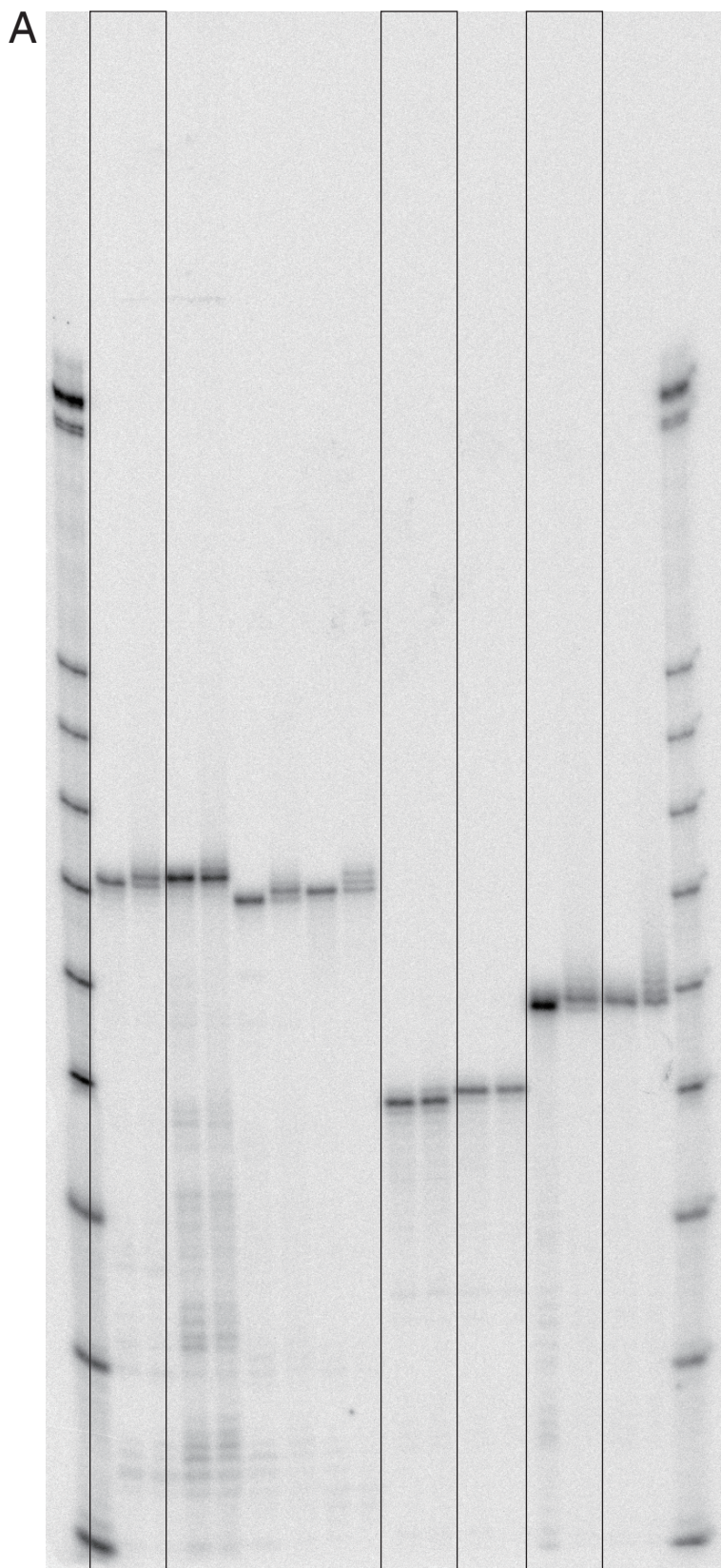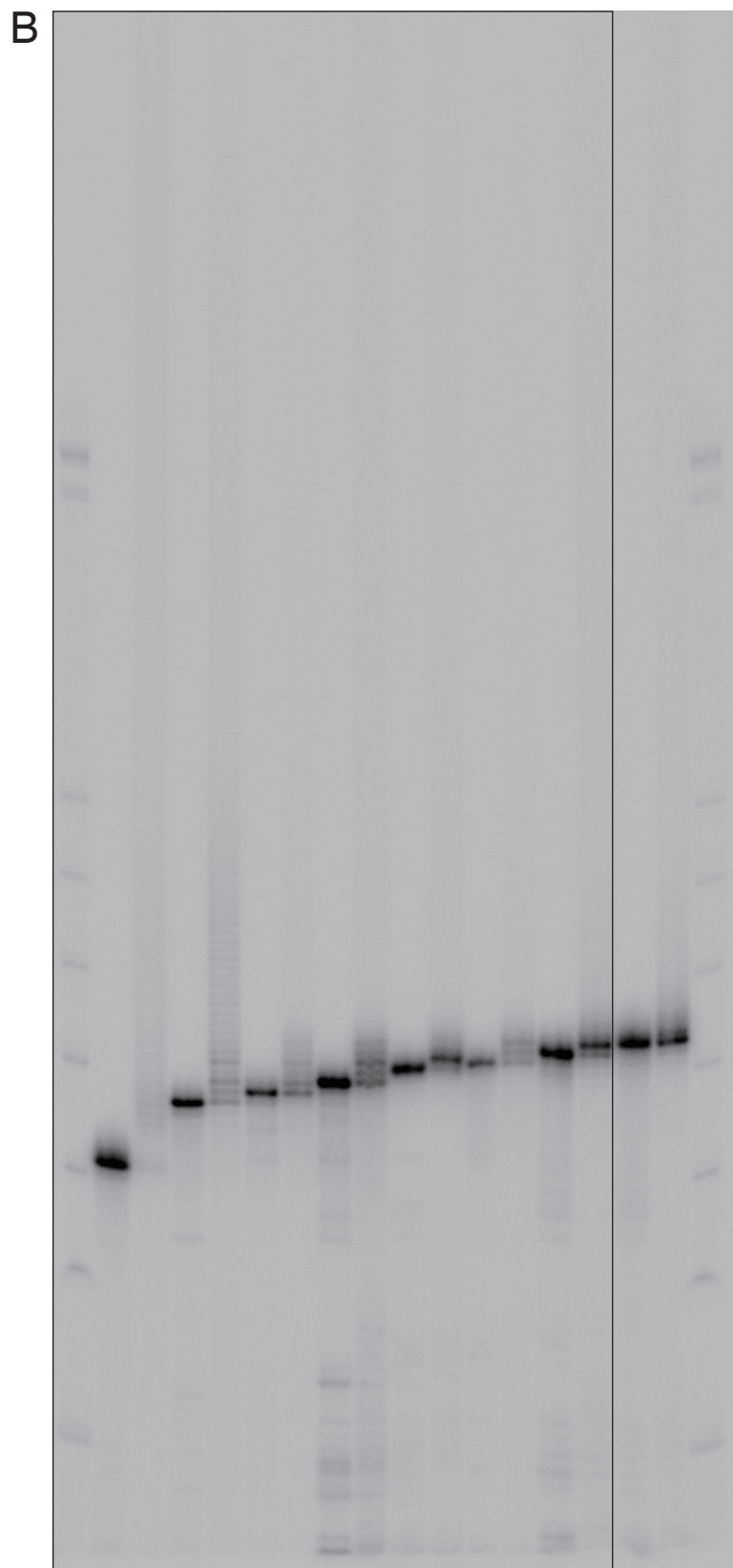

Figure 2 Kim et al

Supplement: Supplementary file 9 [file embj0034-1801-sd9.pdf]
