# Supplementary material for: TUT7 controls the fate of precursor microRNAs by using three different uridylation mechanisms
Source: EMBO J. 2015 May 15;34(13):1801–15. doi: 10.15252/embj.201590931 (PMC4516432; doi:10.15252/embj.201590931)
Supplement: Supplementary file 7 [file embj0034-1801-sd7.pdf]

THE
EMBO
JOURNAL

Manuscript EMBO-2015-90931

TUT7 controls the fate of precursor microRNAs by using three different uridylation mechanisms

Boseon Kim, Minju Ha, Luuk Loeff, Hyeshik Chang, Dhirendra K. Simanshu, Chirlmin Joo, Sisi Li, Mohamed Fareh, Dinshaw J. Patel and V. Narry Kim

Corresponding authors: V. Narry Kim, Seoul National University and Chirlmin Joo, Kavli Institute of NanoScience

Review timeline:

Submission date:	02 January 2015
Editorial Decision:	06 February 2015
Revision received:	11 March 2015
Editorial Decision:	01 April 2015
Revision received:	05 April 2015
Accepted:	08 April 2015

Transaction Report:

(Note: With the exception of the correction of typographical or spelling errors that could be a source of ambiguity, letters and reports are not edited. The original formatting of letters and referee reports may not be reflected in this compilation.)

Editor: Anne Nielsen

1st Editorial Decision 06 February 2015

Thank you for submitting your manuscript for consideration by the EMBO Journal. It has now been seen by three referees whose comments are shown below.

As you will see from the reports, all referees express interest in the findings reported in your manuscript and would support publication in The EMBO Journal, following the clarification/amendment of a number of points. For the revised manuscript, I would particularly encourage you to focus on the following:

-> Please provide additional data on the relative domain contributions and activities seen for truncated and full-length TUT7 (ref#1, p.1, ref#2 p.1, ref #3 p.1)
-> Please include/provide data on the global extent of uridylation and the consequences for mature miRNA accumulation (ref #2, p.2-3)
-> Please clarify the basis for quantifications of miRNA extensions and (if possible) include bulk biochemical assessment of processivity on top of the single-molecule assay (ref#3)
-> I would also ask you to comment/clarify additional minor points raised by the referees.

Given the referees' positive recommendations, I would like to invite you to submit a revised version of the manuscript, addressing the comments of all three reviewers. I should add that it is EMBO

Journal policy to allow only a single round of revision, and acceptance of your manuscript will therefore depend on the completeness of your responses in this revised version.

Thank you for the opportunity to consider your work for publication. I look forward to your revision.

Referee #1:

Kim et al. EMBO J
Jan 2015

Work from this group and others has established that RNA terminal U-transferases (TUTs) have multiple roles in modulating the maturation of miRNAs; addition of oligo(U) tails is associated with pre-miRNA turnover, while mono-uridylation is positively required for the efficient further processing of some pre-miRNAs by Dicer. Two human TUTs seem to play major roles in these processes: TUT4 (ZCCHC11), in association with the RNA-binding protein LIN28A, has been suggested to provide the major oligouridylation activity, while TUT7 (ZCCHC6) appears responsible for the bulk of the monouridylation. These TUTs share extensive sequence (and, presumably, structural) similarity, however, and there is at least some degree of redundancy with respect to both functions. Here, the authors focus on the activity of TUT7 in the absence of LIN28A, and in particular on the ways in which the detailed nature of the pre-miRNA substrate influences the balance between monouridylation / maturation and oligouridylation / turnover.
The data presented indicate that the 3' terminal structure of the pre-miRNA is critical to the outcome of its encounter with TUT7; a single nucleotide 3' overhang is shown to be efficiently monouridylated, in line with an earlier study from this group. However, if the 3' end is further recessed, the outcome is instead oligouridylation. The pre-miRNA loop structure is also shown to be an important determinant of interaction with TUT7. An elegant real-time, single molecule capture approach is used to generate data indicating that TUT7 acts in a distributive manner, whether the outcome is mono- or oligo uridylation, with the pre-miRNA terminal structure governing the frequency of TUT7 binding, but not the dwell time. Finally, the effects of depletion of TUTs on steady-state pre-miRNA / miRNA populations in HeLa cells were determined by selective sequencing. These data support the authors' contention that oligouridylation of 3' trimmed pre-miRNAs is required for their turnover. The consequences of pre-miRNA monouridylation versus oligouridylation were established previously, but the conclusion that RNA substrate structure governs the extent of uridylation, and hence the biological outcome, is likely to be of interest to a wider readership beyond those with specific interests in miRNA metabolism.
The study is characterized by conceptually straightforward but technically challenging experimental approaches and a high overall quality of data and presentation. The authors should nonetheless be encouraged to consider the following points:

1. The characteristics of bacterially produced, truncated recombinant TUT7 are presented in Fig. 1D and Fig. 3, but the remaining in vitro data were generated using FLAG-tagged versions of TUT7 immunopurified from HEK293T cells. LIN28A levels may be low in these cells, but can the authors be sure that the activities seen in vitro are not due, in part at least, to interaction with TUT7-interacting protein(s)? At the very least, it would be useful to have greater clarity in the text about the source of the TUT7 used in vitro.

2. In the siRNA experiements the authors chose to knock down not only TUT4 and TUT7, but also TUT2 (GLD2). The latter has fairly well characterised roles in mRNA and miRNA adenylation, but its possible role in pre-miRNA uridylation in vivo is comparatively poorly defined. Can the authors comment on the likely relative contributions of the three enzymes to the changes in (pre)miRNA abundance described, and particularly the contribution of TUT2 alone?

Referee #2:

In the work by Kim et al., the authors have studied the mechanism by which human TUT7 recognizes and uridylates pre-miRNAs, particularly in the absence of Lin28. As proposed by them in previous work, they find that the overhang of the pre-miRNA is the key structural determinant recognized by TUT7 and its paralogues. In addition to the already described monouridylation activity of TUT7 on group II pre-miRNAs, they describe an apparently more general activity whereby TUT7 adds oligo-U tails on 3' trimmed pre-miRNAs potentially leading to their destabilization. They show that this is a distributive activity where the overhand affects the frequency of interaction between TUT7 and the RNA, ultimately providing a basis for discriminating among different pre-miRNA species. Finally the authors put forward a model to explain the dual roles of uridylation in regulating the fate of pre-miRNAs.

Overall, this is an interesting study showing a potentially more general (and complex) role for TUTs in regulating the fate of pre-miRNAs than previously conceived. However, as described below, some of the conclusions require support from further experimentation.

Major points:

1. In Figure 1, the authors conclude that the C-terminal domains of TUT7 (including the catalytic motif), but not its N-terminal half, are required for pre-miRNA mono-uridylation. In fact, they perform this analysis exclusively in the context of mono-uridylation of group I vs group II let-7 pri-miRNAs. Since one of the main claims of this work concerns a new oligo-uridylation activity for TUT7, the authors should test the domain dependence in the oligo-uridylation on pre-miRNA species such as 3' truncated pre-miRNAs (Ac-pre or extense 5' overhangs). Are there additional interaction partners required for pre-miRNA oligo-uridylation in the absence of Lin28? For instance, the authors could check whether the recombinant TUT7 (rTUT7) can mediate this effect.

2. In Figure 4 the authors analyse the uridylation patterns of group I and II pre-let miRNAs, confirming their previous results about mono-uridylation. They further analyze 54 additional pre-miRNAs where they detect increased trimmed species accumulation upon TUT7/4/2 knock down. Within the trimmed species they observe decreased uridylation upon TUT7/4/2 knock down, suggesting a general activity of TUT7/4/2 on a broader range of pre-miRNAs. The authors should show (possibly as an additional panel in Fig. 4d) the uridylation activity not only on trimmed but also on total pre-miRNAs to give an idea of the relative uridylation efficiencies in both populations. These data are perhaps hidden in the Supplementary Tables accompanying the manuscript. Are pre-miRNAs with higher uridylation efficiency enriched for miRNAs reported to produce ac-pre-miRNA?

3. In Figure 4 d-e, the authors conclude that TUT7/4/2 uridylates 3'trimmed pre-miRNAs in general, and that this activity might lead to destabilization of defective pre-miRNAs. Based in this idea, one could therefore predict that TUT7/4/2 knock down, by increasing the accumulation of defective pre-miRNAs, would increase the levels of mature (perhaps also defective) miRNAs. However, according to a recent reports (e.g., 2014 Gregory's work in NAR) mature miRNA levels are not affected by TUT7/4 depletion in HeLa cells, which would go against the authors' suggestion. The authors should therefore measure mature miRNA levels and mature miRNA modification (e.g. U-tailing) upon TUT7/4/2 knock down in their experimental conditions.

Minor points:

1. In Figure 1b the time of reaction is not indicated making it difficult to compare with Figure 1d.

2. On page 11, second line, the authors refer to Pre-let-7e as a group II pre-miRNA while it belongs to group I.

3. Data in Fig. 2b, bottom panel should be better discussed in the text. For example, there seems to be an abrupt change in average U-tail length between substrates having 3-nt versus 4-nt 5' overhang. On the other hand, the 1 nt 5' overhang substrate seems to be extended by just one U residue (making it blunt) while when one start with a blunt end substrate, the addition of Us seems to be more effective.

4. Fig. S2b. Kinetic data indicate that at later time-point (5 min) recombinant TUT7 actually adds more than one U residue to the unmodified substrate. Is this time effect more general? Please comment.

5. p. 7 bottom (and also methods). "Passivated" surface. I am not aware of the existence of this word in English. Why not to use more descriptive way and write that the surface was PEG-ylated for this and that purpose.

Referee #3:

Uridylation of pre-miRNAs by terminal uridylyl transferase represents an important layer of regulation on miRNA biogenesis and function. Studies in mammalian cells have revealed that TUT2/4/7 function together with Lin28 to oligo-uridylate pre-miRNAs, inhibiting dicer processing and miRNA functions. One the other hand, in the absence of Lin28, TUT2/4/7 are able to mono-uridylate group II pre-miRNAs and the addition of U promotes dicer processing. In this study, the authors combined in vitro biochemical and deep sequencing approaches to examine the molecular mechanism of TUT2/4/7-mediated mono-uridylation of pre-miRNAs with a focus on TUT7. They first delineate the functional domains of TUT7 for mono-uridylation by testing the activity of truncated proteins. Further the authors determined that the terminal loop and 3' end structure pre-let7a-1 are two key elements recognized by TUT7 for uridylation. Moreover, by making structural variants of pre-let7a-1 the authors observed that TUT7 was able to oligo-uridylate blunt end pre-let7a-1 as well as 3' trimmed pre-let71-1 variants. Very nicely the single molecule study demonstrated that the oligo- and mono-uridylation activity of TUT7 on pre-let7a-1 (wild type and mutants) is distributive. By employing deep sequencing method the authors were able to confirm that TUT2/4/7 are responsible for majority of mono-uridylation of group II pre-let7 and the degradation of 3' trimmed pre-miRNAs by oligo-uridylation.

Overall, the manuscript is well written and the data presented are interesting. However, there are some issues need to be addressed, please see specific comments below.

Major concerns:

• In figure 1d and sup Fig2a it seems that rTUT7 is able to oligo-uridylate pre-let7a-1 after 5min incubation, even though the IP Flag-TUT7 does not. Why is it? Does the recombinant truncated protein have different properties from the full length protein? Since the reaction time is crucial for the enzyme activity, the reaction time should be labeled clearly in the figure or stated in the figure legend including the enzymatic activity assays with immuno-purified TUTs.

• It is not very clear how are the average U tail lengths calculated in fig 2b? It looks like lane 4 has a longer U tail than lane 2. In particular, with ΔCUUUC looks like relatively even bands up to 90+ nt (>20U), but the average is cited as only 6U. Other numbers also do not visually appear to be like the gel. For example, the unmodified substrate is listed as 1.31U, which fits with the substrate mostly getting 1nt larger, but ΔUUC is listed as 1.43U, even though there is an even distribution of multiple bands.

• An unexpected result is the conclusion that TUT7 is distributive even on the long tails show with ac-pre. Actually, the long tails on gels are much more visible with ΔCUUUC (Fig 2b) so this may provide a better substrate to test this with single molecule assay (unless I missed that this is shown in the supplements). I think it would be nice to perform a more conventional assay with ensemble measurements to support this conclusion. As I understood, a processive enzyme will continue to modify its substrate following dilution of the reaction, whereas the action of a distributive enzyme will be inhibited since it has to rebind the substrate. Since they have robust extension of ac-pre or ΔCUUUC, I think it should be able to find a time-course to show this in an ensemble measurement.

Is there any interpretation for the dwell time of pre-let7a-1 L4 being longer than that of the unmodified pre-let7a-1 (Fig 3d)?

© European Molecular Biology Organization

• Related to this, I am confused about the binding frequency experiment. When the unmodified pre-let7a-1 is mixed with pre-let7a-1 variants, the authors show that rTUT7 prefers to bind the 3' trimmed pre-let7a-1. However, for the in vitro biochemical assays, rTUT7 is incubated with only one substrate, why rTUT7 displays mono- vs. oligo-uridylation activity on different substrate? When rTUT7 oligo-uridylate 3' trimmed variants, there must be an intermediate state when these variants have a 2nt 3' overhang, why doesn't rTUT7 stop there or fall off?

Minor concerns:
• The evidence for the requirement of C-terminal domains for TUT7 mono-uridylation activity is not very clear (Fig 1b). The fact that NP alone is inactive could be caused by necessity of all three zinc-finger domains at the C-terminus, and/or improper folding of this truncated protein. It may be useful to test ΔPAP1 with point mutations of ZF. This is maybe a lower priority for this study, but interesting.

• Apparently, the data from supp 2d shows that TUT2 doesn't discriminate between pre-let7a-1 variants bearing different overhang structures. So the statement that 'the primary cis-acting element recognized commonly by TUT7/4/2 is the overhang structure of pre-miRNA' does not seem reflecting this. Is the terminal loop of pre-let7a-1 is important for TUT7 recognition but not for TUT4/2? Then what could be the features recognized by TUT2? Moreover, it seems that TUT4/2 display oligo- than mono-uridylation activity even with unmodified pre-let7a-1.

• It would be nice to have the SDS-gel to show the purified rTUT7.

• Pre-let7e should be group I instead of group II?

• It is confusing to see Koffs listed in supp fig3b since the ΔΔG is calculated with Kons.

1st Revision - authors' response 11 March 2015

Response to Reviewers

We thank the reviewers for carefully reading our manuscript and providing constructive feedback. We hope that the reviewers find our responses clear and to the point.

Referee #1

Major points:

1. The characteristics of bacterially produced, truncated recombinant TUT7 are presented in Fig. 1D and Fig. 3, but the remaining in vitro data were generated using FLAG-tagged versions of TUT7 immunopurified from HEK293T cells. LIN28A levels may be low in these cells, but can the authors be sure that the activities seen in vitro are not due, in part at least, to interaction with TUT7-interacting protein(s)? At the very least, it would be useful to have greater clarity in the text about the source of the TUT7 used in vitro.

- For all pre-let-7a-1 mutants that we generated, we carried out in vitro experiments both with immunopurified full-length TUT7 (Fig 2) and with recombinant TUT7 951-1495 (rTUT7) (Fig 2 and Fig E2A and B). rTUT7 has the same substrate preference as the immunopurified full-length TUT7, indicating that TUT7 does not require any additional cofactor for pre-miRNA mono-uridylation and trimmed pre-miRNA oligo-uridylation. As suggested, we clearly indicate the source of the TUT7 used in vitro in the text (p. 6-8).

- We have previously shown that TUT4 stably interacts with Lin28 only in the presence of let-7 pre-miRNAs (Heo et al, 2009). As the pre-let-7 expression level is very low in HEK293T cells (Heo et al, 2008), our immunopurified TUT7 from HEK293T cells does not co-purify with Lin28. We immunopurified flag-TUT7 protein to near homogeneity as we demonstrated in our recent paper (Lim et al, 2014).

2. In the siRNA experiments the authors chose to knock down not only TUT4 and TUT7, but also TUT2 (GLD2). The latter has fairly well characterised roles in mRNA and miRNA adenylation, but its possible role in pre-miRNA uridylation in vivo is comparatively poorly defined. Can the authors comment on the likely relative contributions of the three enzymes to the changes in (pre)miRNA abundance described, and particularly the contribution of TUT2 alone?

- Following the suggestion, we now discuss the relative contributions of the three enzymes in the text (p.14). Briefly, TUT7 and TUT4 are highly similar to each other, and participate in oligo-uridylation of trimmed pre-miRNAs, mono-uridylation of group II pre-miRNAs, and Lin28-mediated oligo-uridylation (Fig 5A). The contribution of TUT2 appears to be limited to mono-uridylation of group II pre-miRNAs.

Referee #2

Major points:

1. In Figure 1, the authors conclude that the C-terminal domains of TUT7 (including the catalytic motif), but not its N-terminal half, are required for pre-miRNA mono-uridylation. In fact, they perform this analysis exclusively in the context of mono-uridylation of group I vs group II let-7 pri-miRNAs. Since one of the main claims of this work concerns a new oligo-uridylation activity for TUT7, the authors should test the domain dependence in the oligo-uridylation on pre-miRNA species such as 3′ truncated pre-miRNAs (Ac-pre or extense 5′ overhangs). Are there additional interaction partners required for pre-miRNA oligo-uridylation in the absence of Lin28? For instance, the authors could check whether the recombinant TUT7 (rTUT7) can mediate this effect.

- Actually, we already provided the result of the suggested experiment (in vitro uridylation assay of 3′ trimmed pre-miRNAs with recombinant TUT7 951-1495) in the original submission (Please see Fig E2B). The result indicates that the C-term half of TUT7 is sufficient for oligo-uridylation activity without requiring any cofactor.

2. In Figure 4 the authors analyse the uridylation patterns of group I and II pre-let miRNAs, confirming their previous results about mono-uridylation. They further analyze 54 additional pre-miRNAs where they detect increased trimmed species accumulation upon TUT7/4/2 knock down. Within the trimmed species they observe decreased uridylation upon TUT7/4/2 knock down, suggesting a general activity of TUT7/4/2 on a broader range of pre-miRNAs. The authors should show (possibly as an additional panel in Fig. 4d) the uridylation activity not only on trimmed but also on total pre-miRNAs to give an idea of the relative uridylation efficiencies in both populations. These data are perhaps hidden in the Supplementary Tables accompanying the manuscript. Are pre-miRNAs with higher uridylation efficiency enriched for miRNAs reported to produce ac-pre-miRNA?

- The requested information (uridylation on total pre-miRNAs) was presented in previous Supplementary Fig. 4B. We apologize for unclear presentation in the original submission. To increase visibility, we modified the previous Supplementary Fig. 4B and now show it as Fig 4B.
- Pre-miRNAs with higher uridylation efficiency are not enriched for miRNAs reported to produce ac-pre-miRNAs. But please note that because uridylated pre-miRNAs are subject to decay, the steady state levels of uridylated pre-miRNAs will be determined not only by uridylation rates but also by decay rates.

3. In Figure 4 d-e, the authors conclude that TUT7/4/2 uridylates 3′ trimmed pre-miRNAs in general, and that this activity might lead to destabilization of defective pre-miRNAs. Based in this idea, one could therefore predict that TUT7/4/2 knock down, by increasing the accumulation of defective pre-miRNAs, would increase the levels of mature (perhaps also defective) miRNAs. However, according to a recent reports (e.g., 2014 Gregory's work in NAR) mature miRNA levels are not affected by TUT7/4 depletion in HeLa cells, which would go against the authors' suggestion. The authors should therefore measure mature miRNA levels and mature miRNA modification (e.g. U-tailing) upon TUT7/4/2 knock down in their experimental conditions.

- In fact, we and others have already shown that the levels of mature miRNAs (with an exception of the let-7 family) are not significantly affected by TUT7/4/2 knockdown (Heo et al, 2012; Liu et al, 2014; Thornton et al, 2012). This is expected because trimmed pre-miRNAs are not processed by Dicer due to their aberrant overhang structures, and they are subject to decay without contributing to mature miRNA production (Park et al, 2011). So the lack of changes in mature miRNA levels is actually consistent with our model. This point is now discussed in the text (p.13) to avoid confusion.

Minor points:

1. In Figure 1b the time of reaction is not indicated making it difficult to compare with Figure 1d.

- Now we provide the time of reaction in figure legends.

2. On page 11, second line, the authors refer to Pre-let-7e as a group II pre-miRNA while it belongs to group I.

- We have corrected this typo.

3. Data in Fig. 2b, bottom panel should be better discussed in the text. For example, there seems to be an abrupt change in average U-tail length between substrates having 3-nt versus 4-nt 5′ overhang. On the other hand, the 1 nt 5′ overhang substrate seems to be extended by just one U residue (making it blunt) while when one start with a blunt end substrate, the addition of Us seems to be more effective.

- As the reviewer correctly pointed out, RNAs containing a long 5′ overhang (≥ 4 nt 5′ overhang, Ac-pre and ΔCUUUC) were more strongly oligo-uridylated than those with a short 5′ overhang. Although there are differences in the uridylation activities of TUTs depending on various pre-let-7a-1 mutants, the exact reason for this differential activities are not clear at this point.

4. Fig. S2b. Kinetic data indicate that at later time-point (5 min) recombinant TUT7 actually adds more than one U residue to the unmodified substrate. Is this time effect more general? Please comment.

- When the reaction is prolonged, multiple rounds of distributive "mono"-uridylation occurs, which results in apparent "oligo"-uridylation. This point is now explained in the text (p. 6 and 15).

5. p. 7 bottom (and also methods). "Passivated" surface. I am not aware of the existence of this word in English. Why not to use more descriptive way and write that the surface was PEG-ylated for this and that purpose.

- We have replaced the term "passivated" with a more specified term "PEGylated".

Referee #3

Major points:

1. In figure 1d and sup Fig2a it seems that rTUT7 is able to oligo-uridylate pre-let7a-1 after 5min incubation, even though the IP Flag-TUT7 does not. Why is it? Does the recombinant truncated protein have different properties from the full length protein? Since the reaction time is crucial for the enzyme activity, the reaction time should be labeled clearly in the figure or stated in the figure legend including the enzymatic activity assays with immuno-purified TUTs.

- The recombinant TUT7 951-1495 (rTUT7) protein has the same substrate preference as the immunopurified full-length TUT7 protein (Fig 2 and Fig E2A and B). But the amount of rTUT7 used in Fig 1D and Fig E2A was larger than that of immunopurified full-length TUT7 in Figs 1A and 2. The apparent "oligo"-uridylation observed in the rTUT7 assay resulted from multiple cycles of distributive mono-uridylation. This point is now explained in the text to avoid confusion (p. 6 and 15).
- As suggested, now we provide the reaction time for all in vitro assays in figure legends.

2. It is not very clear how are the average U tail lengths calculated in fig. 2b? It looks like lane 4 has a longer U tail than lane 2. In particular, with ΔCUUUC looks like relatively even bands up to 90+ nt (>20U), but the average is cited as only 6U. Other numbers also do not visually appear to be like the gel. For example, the unmodified substrate is listed as 1.31U, which fits with the substrate mostly getting 1nt larger, but ΔUUC is listed as 1.43U, even though there is an even distribution of multiple bands.

- We apologize for not describing the method in the original submission. Now we provide the method for quantification of in vitro uridylation data in the expanded view. Please note that it is difficult to precisely quantify the average U-tail length because the signal is widely distributed along the long lane on sequencing gel (20x40 cm, 0.4 mm thick). Although we feel that this semi-quantitative measure is still useful, we will remove the numbers from Fig 2B if the reviewers feel the presented numbers are confusing.

3. An unexpected result is the conclusion that TUT7 is distributive even on the long tails show with ac-pre. Actually, the long tails on gels are much more visible with ΔCUUUC (Fig. 2b) so this may

provide a better substrate to test this with single molecule assay (unless I missed that this is shown in the supplements).

- The reviewer is right in that ΔCUUUC is a better substrate. In fact, the substrate we used in single molecule assay was ΔCUUUC but was mistakenly written as ΔUUUC. We have corrected the manuscript and the figures (Figs 3F and G, and Fig E3A and B).

I think it would be nice to perform a more conventional assay with ensemble measurements to support this conclusion. As I understood, a processive enzyme will continue to modify its substrate following dilution of the reaction, whereas the action of a distributive enzyme will be inhibited since it has to rebind the substrate. Since they have robust extension of ac-pre or ΔCUUUC, I think it should be able to find a time-course to show this in an ensemble measurement.

- As suggested, we carried out ensemble measurements with dilution, and present the data in Expanded View Figure 4C. Briefly, in the presence of Lin28b, rTUT4 (267-1312) oligo-uridylates unmodified pre-let-7a-1, and this is not affected by dilution, suggesting that Lin28-mediated oligo-uridylation is indeed processive as we previously reported (Yeom et al, 2011). However, oligo-uridylation of ac-pre-let-7a-1 was repressed strongly after dilution. Thus, without Lin28, TUT4 acts in a distributive manner, which supports our conclusion from single molecule experiments (Fig 3).

Is there any interpretation for the dwell time of pre-let7a-1 L4 being longer than that of the unmodified pre-let7a-1 (Fig. 3d)?

- We speculate that the loop might also play a role in the release of the substrate. However, without any structural information (e.g. a crystal structure), it is difficult to further speculate. In the revised manuscript, we have provided a short description on this observation but have made it very short (p.9).

4. Related to this, I am confused about the binding frequency experiment. When the unmodified pre-let7a-1 is mixed with pre-let7a-1 variants, the authors show that rTUT7 prefers to bind the 3' trimmed pre-let7a-1. However, for the in vitro biochemical assays, rTUT7 is incubated with only one substrate, why rTUT7 displays mono- vs. oligo-uridylation activity on different substrate? When rTUT7 oligo-uridylate 3' trimmed variants, there must be an intermediate state when these variants have a 2nt 3' overhang, why doesn't rTUT7 stop there or fall off?

- The reviewer correctly pointed out that rTUT7 tends to stop uridylation after producing a 2 nt 3′ overhang. The figure below shows the uridylation products of pre-let-7a-1 ΔC in a time course experiment. While pre-let-7a-1 ΔC (71 nt) and mono-uridylated pre-let-7a-1 ΔC (72 nt) are quickly uridylated (lanes 2-3), RNA with a 2 nt 3′ overhang (73 nt, marked with a red arrowhead) is not readily uridylated and accumulated, indicating that rTUT7 cannot re-bind to the RNA with a 2 nt 3′ overhang efficiently. We also observed this phenomenon in uridylation assays with unmodified pre-let-7a-1 or mutants with a short 5′ overhang (ΔC, ΔUC, ΔUUC, ΔUUUC) (Fig 2B and Fig E2B and D).

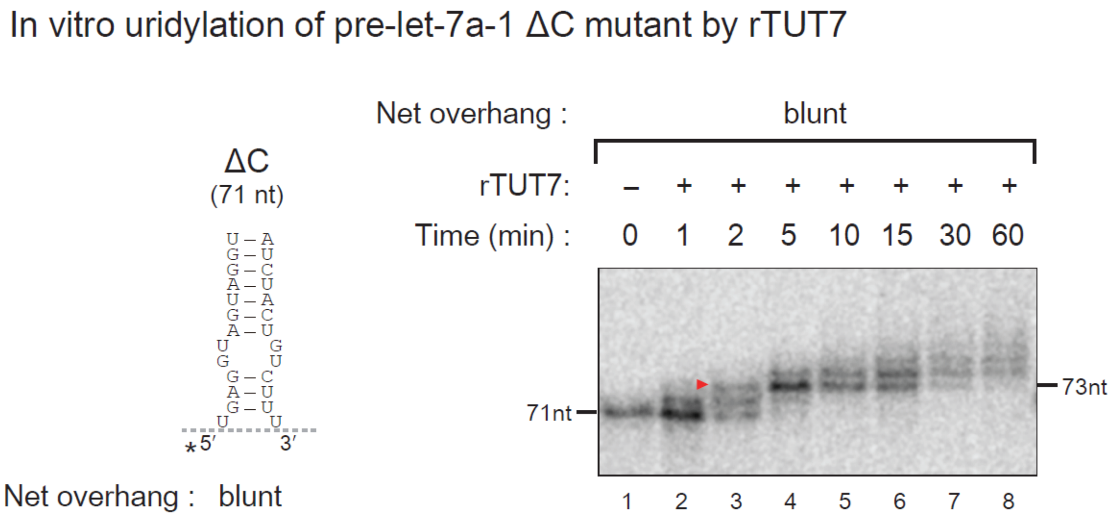

Minor points:

1. The evidence for the requirement of C-terminal domains for TUT7 mono-uridylation activity is not very clear (Fig. 1b). The fact that NP alone is inactive could be caused by necessity of all three zinc-finger domains at the C-terminus, and/or improper folding of this truncated protein. It may be useful to test ΔPAP1 with point mutations of ZF. This is maybe a lower priority for this study, but interesting.

- As the reviewer pointed out, there are two possibilities which can explain why NP mutant lost its activity. Now we mention these possible reasons in the text (p.6). We hope that the reviewer will agree with us in that further mutagenesis experiments on multiple ZF will be out of the scope of this manuscript.

2. Apparently, the data from supp 2d shows that TUT2 doesn't discriminate between pre-let7a-1 variants bearing different overhang structures. So the statement that 'the primary cis-acting element recognized commonly by TUT7/4/2 is the overhang structure of pre-miRNA' does not seem reflecting this. Is the terminal loop of pre-let7a-1 is important for TUT7 recognition but not for TUT4/2? Then what could be the features recognized by TUT2?

- We showed the data that only TUT7 recognizes both terminal loop and 3′ overhang structure while TUT4 and TUT2 recognize only 3′ overhang structure (Fig 2 and Fig E2C and D). We have previously shown that TUT7/4/2 act specifically on pre-miRNAs with a 1 nt 3′ overhang structure (Heo et al, 2012). Thus, TUT2 recognizes the 1 nt 3′ overhang structure while it does not prefer pre-miRNAs with a long 5′ overhang (pre-let-7a-1 ΔCUUUC and Ac-pre).

Moreover, it seems that TUT4/2 display oligo- than mono-uridylation activity even with unmodified pre-let7a-1.

- The oligo-uridylation activity results from multiple cycles of distributive mono-uridylation. This is observed when a large amount of TUT enzyme is used or when reaction time is extended. This point is now clarified in the text (p. 6 and 15).

3. It would be nice to have the SDS-gel to show the purified rTUT7.

- This was provided in our recent publication (Lim et al, 2014).

4. Pre-let7e should be group I instead of group II?

- We have corrected this typo.

5. It is confusing to see Koffs listed in supp fig3b since the ΔΔG is calculated with Kons.

- We have included the relative Kons in the table.

We appreciate the insightful comments and constructive suggestions.

REFERENCES

Heo I, Ha M, Lim J, Yoon MJ, Park JE, Kwon SC, Chang H, Kim VN (2012) Mono-uridylation of pre-microRNA as a key step in the biogenesis of group II let-7 microRNAs. Cell 151: 521-532

Heo I, Joo C, Cho J, Ha M, Han J, Kim VN (2008) Lin28 mediates the terminal uridylation of let-7 precursor MicroRNA. Molecular cell 32: 276-284

Heo I, Joo C, Kim YK, Ha M, Yoon MJ, Cho J, Yeom KH, Han J, Kim VN (2009) TUT4 in concert with Lin28 suppresses microRNA biogenesis through pre-microRNA uridylation. Cell 138: 696-708

Lim J, Ha M, Chang H, Kwon SC, Simanshu DK, Patel DJ, Kim VN (2014) Uridylation by TUT4 and TUT7 Marks mRNA for Degradation. Cell 159: 1365-1376

Liu X, Zheng Q, Vrettos N, Maragkakis M, Alexiou P, Gregory BD, Mourelatos Z (2014) A MicroRNA Precursor Surveillance System in Quality Control of MicroRNA Synthesis. Molecular cell 55: 868-879

Park JE, Heo I, Tian Y, Simanshu DK, Chang H, Jee D, Patel DJ, Kim VN (2011) Dicer recognizes the 5' end of RNA for efficient and accurate processing. Nature 475: 201-205

Thornton JE, Chang HM, Piskounova E, Gregory RI (2012) Lin28-mediated control of let-7 microRNA expression by alternative TUTases Zcchc11 (TUT4) and Zcchc6 (TUT7). RNA 18: 1875-1885

Yeom KH, Heo I, Lee J, Hohng S, Kim VN, Joo C (2011) Single-molecule approach to immunoprecipitated protein complexes: insights into miRNA uridylation. EMBO Rep 12: 690-696

2nd Editorial Decision 01 April 2015

Thank you for submitting the revised version of your manuscript to The EMBO Journal. The study has now been seen by two of the original referees (comments included below) and as you will see they both support publication. I am therefore happy to let you know that your study is in principle ready for acceptance here.

However, before we officially accept your study and transfer your manuscript files for production I have to ask you to address the following editorial points in a final revised version of your manuscript:

-> We generally require that all information relevant to the main experiments in the manuscript should be included in Materials and Methods. I would therefore ask you to move the supplemental materials (at least the experimental sections) into the main manuscript file.

-> Please comment on the one remaining concern raised by referee #3.

-> Please also consider changing title and abstract along the following lines:
'TUT7 controls the fate of precursor microRNAs by uridylation'

Thank you again for your contribution to The EMBO Journal, I look forward to receiving the final version.

Referee #2:

I am happy with the revised version of MS

Referee #3:

I think the current manuscript is suitable for publication, and congratulate the authors on a nice study. However, they still didn't address the question why TUT7 didn't stop uridylation after the ac-pre-let-7a-1 is modified to the state where it bears 2nt 3' overhang (major point 4).

2nd Revision - authors' response 05 April 2015

Response to Reviewers

We appreciate the reviewers for the positive comments on the revised manuscript.

Referee #3: I think the current manuscript is suitable for publication, and congratulate the authors on a nice study. However, they still didn't address the question why TUT7 didn't stop uridylation after the ac-pre-let-7a-1 is modified to the state where it bears 2nt 3' overhang (major point 4).

- The exact answer to the above question is not clear at this point. Our working hypothesis is the following.
- With pre-miRNA substrates that are trimmed by ≤ 4 nt (ΔC, ΔUC, ΔUUC, ΔUUUC), we indeed observed that distributive uridylation stops when the substrates obtain a 2 nt 3′ overhang (Fig 2B). Note that four nucleotides from the 3′ end of unmodified pre-let-7a-1 consist predominantly of uridines. Thus, uridylation can partially restore trimmed nucleotides that were originally present. Consequently, trimmed pre-let-7a-1 recovers its original secondary structure and the uridylation ends with the 2 nt 3′ overhang (Fig R1, lower gray box).
- However, when the substrates are trimmed by ≥ 5 nt (ΔCUUUC and Ac-pre), uridylation cannot restore the double stranded stem structure of the unmodified pre-let-7a-1 (Fig R1, lower white box). The resulting single stranded U-tails of ΔCUUUC and Ac-pre can be continuously uridylated without stopping (Fig 2B).

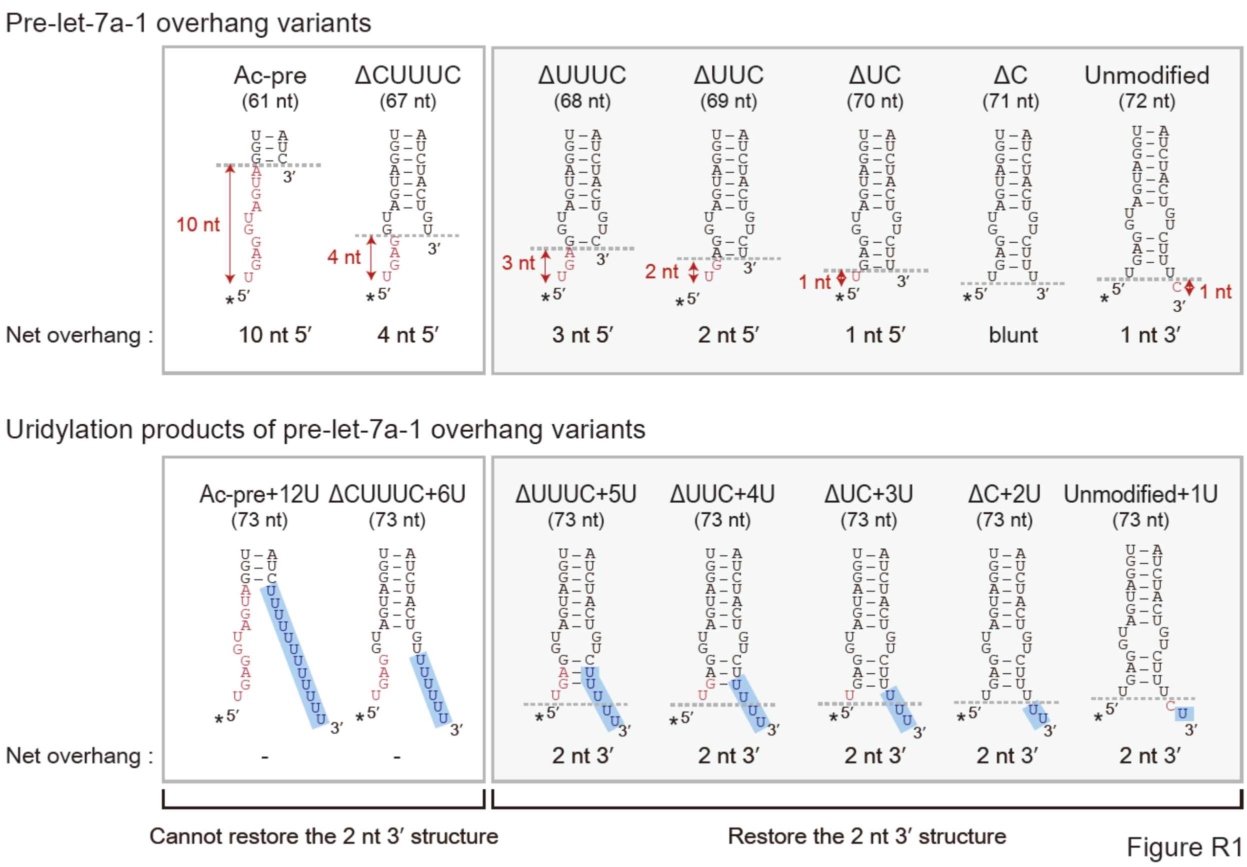

Figure R1

3rd Editorial Decision 08 April 2015

Thank you for submitting the final revision of your manuscript, I am pleased to inform you that your study has now been accepted for publication in the EMBO Journal.
